# Mapping protein direct interactome of oxidoreductases with small molecular chemical cross-linkers in live cells

**DOI:** 10.1016/j.redox.2023.102642

**Published:** 2023-02-24

**Authors:** Ting Wu, Shang-Tong Li, Yu Ran, Yinuo Lin, Lu Liu, Xiajun Zhang, Lianqi Zhou, Long Zhang, Donghai Wu, Bing Yang, Shibing Tang

**Affiliations:** a Zhejiang Provincial Key Laboratory for Cancer Molecular Cell Biology, Life Sciences Institute, Zhejiang University, Hangzhou, 310058, China; b Cancer Center, Zhejiang University, Hangzhou, 310058, China; c Glbizzia Biosciences Co., Ltd, Beijing, 102601, China; d Center for Chemical Biology and Drug Discovery, Guangzhou Institutes of Biomedicine and Health, Chinese Academy of Sciences, Guangzhou, 510530, China; e China-New Zealand Joint Laboratory on Biomedicine and Health, Guangzhou Institutes of Biomedicine and Health, Chinese Academy of Sciences, Guangzhou, 510530, China

## Abstract

Identifying direct substrates of enzymes has been a long-term challenge. Here, we present a strategy using live cell chemical cross-linking and mass spectrometry to identify the putative substrates of enzymes for further biochemical validation. Compared with other methods, our strategy is based on the identification of cross-linked peptides supported by high-quality MS/MS spectra, which eliminates false-positive discoveries of indirect binders. Additionally, cross-linking sites allow the analysis of interaction interfaces, providing further information for substrate validation. We demonstrated this strategy by identifying direct substrates of thioredoxin in both *E. coli* and HEK293T cells using two bis-vinyl sulfone chemical cross-linkers BVSB and PDES. We confirmed that BVSB and PDES have high specificity in cross-linking the active site of thioredoxin with its substrates both *in vitro* and in live cells. Applying live cell cross-linking, we identified 212 putative substrates of thioredoxin in *E. coli* and 299 putative S-nitrosylation (SNO) substrates of thioredoxin in HEK293T cells. In addition to thioredoxin, we have shown that this strategy can be applied to other proteins in the thioredoxin superfamily. Based on these results, we believe future development of cross-linking techniques will further advance cross-linking mass spectrometry in identifying substrates of other classes of enzymes.

## Highlights

• Capturing protein interactions of enzymes and their direct binding proteins or potential substrates in live cells.
• Development of new cysteine and lysine targeted chemical cross-linkers.
• Enrichment of inter-protein cross-linked peptides with two-step purification and SCX enrichment.

## 1 Introduction

Thioredoxin (Trx) is an oxidoreductase essential to life as it has been known to support ancient life forms dating back ∼4 billion years [1]. Ubiquitously expressed in all organisms, Trx protects proteins from oxidative damage, helps cells resist environmental stresses, catalyzes denitrosylation and transnitrosylation to regulate cellular signaling, and is also involved in the inflammatory response [2]. In addition to Trx, proteins in the Trx superfamily, sharing a CxxC sequence motif and a conserved Trx fold, also play important roles in maintaining cellular redox balance by scavenging reactive oxygen species, thus they are important biomarkers of various diseases [3]. For example, the imbalance of redox homeostasis has been shown to cause heart injury [4]. To get a comprehensive understanding of Trx superfamily proteins in normal physiology and pathology, it is critical to identify interacting proteins and/or substrates of these proteins.

A disulfide trapping method utilizing a Trx1_W**C**GP**A**_ mutant has been applied to map interacting proteins of Trx1, resulting in the identification of 201 new Trx1 substrates [5]. This method traps reaction intermediates during reduction by forming inter-protein disulfide bonds. However, the endogenous wild type Trx1 is not present in the experiments, which could cause artifacts due to the imbalance of redox homeostasis. Quantitative mass spectrometry analyses with or without stable isotopic labeling were also used to identify Trx targets [6,7]. However, these methods cannot differentiate direct or indirect targets of Trxs. Genetically encoded chemical cross-linkers (GECXs) can spontaneously capture weak and instantaneous protein-protein interactions in live cells [[8], [9], [10], [11]]. Electrophilic unnatural amino acids (Uaas), such as BprY and FSK, have been used to capture direct binding proteins of Trx1 [[10], [11], [12]], and 205 direct binding proteins of Trx1 were identified by incorporating BprY into Trx1 in *E. coli* [12]. In GECXs, the incorporation of unnatural amino acids into target proteins requires the co-expression of orthogonal aminoacyl-tRNA synthetase/tRNA pair in live cells. So far, this method has only been applied to study Trx1 in *E. coli*, but has not been successfully implemented in mammalian cells.

Cross-linking Mass Spectrometry (XL-MS) is a powerful tool in the studies of protein structures and protein-protein interactions [13,14]. Recently, significant progress has been made in live cells cross-linking to map protein interactome in a whole-proteome scale [15,16]. Affinity Purification-Mass Spectrometry coupled with chemical cross-linking is also useful to map direct protein-protein interactions and their interaction interfaces [[17], [18], [19]]. Previously, a cross-linker DVSF was applied to trap the interactions of oxidoreductase and its binding proteins in live cells [[20], [21], [22]]. This strategy has been validated by several oxidoreductases and their known substrates. However, few cross-linked peptides were available to support direct enzyme-substrate binding, and comprehensive identification of substrates in an unbiased fashion was not achieved.

In this study, two robust vinyl sulfone embedded cross-linkers named BVSB and PDES were developed to comprehensively capture and identify protein direct interactome of oxidoreductases in live cells. With cross-linked peptides supported by high-quality MS/MS spectra, we are confident that we identified direct binders. Applying this method to *E. coli*, we have successfully identified 212 putative Trx1 substrates in *E. coli*. We next performed cross-linking in HEK293T cells to identify 299 putative SNO substrates of TXN1. Finally, we have demonstrated that this method can be broadly applied to study other protein members in the Trx superfamily.

## 2 Materials and methods

### 2.1 Protein expression

pET-22b-Trx1-6 × His, pET-22b-Tpx-Flag-6 × His, pET-22b-PAPR-Flag-6 × His, pRSF-Duet1-Trx1-6 × His-Tpx-Flag, pRSFDuet-1-Trx1-6 × His-PAPR-Flag and pET-22b-N-terminal-6 × His-Trx1K4R–K19R were transformed into BL21(DE3) chemical competent cells, respectively. The transformants were plated on an LB agar plate with antibiotics and incubated overnight at 37°C. A single colony was inoculated into 5 mL of 2 × YT with antibiotics and cultured overnight at 37°C. A volume of 1 mL cell culture was diluted into 100 mL LB with antibiotics and shaken vigorously. The cell culture was induced with 400 μmol IPTG (Sangon) when OD 600 reached about 0.6, and then incubated at 37°C for 4 h (pET-22b-Trx1-6 × His, pET-22b-Tpx-Flag-6 × His and pET-22b-PAPR-Flag-6 × His) and 2 h (co-expression plasmids and 2 KR mutant plasmids) respectively. Cell pellets were collected by centrifugation at 4000 × *g* for 30 min at 4°C, and stored at −80°C for purification or for BVSB/PDES cross-linking.

### 2.2 Cell culture and transfection

Human 293T cell was grown in DMEM supplemented with 10% FBS and antibiotics, and was cultured in a 37°C, 5.0% CO2 incubator. For transient transfection, 15 μg TXN1-6 × His or TRP14-6 × His mammalian expression vectors and 30 μL PEI 25K (BIOHUB) were mixed with 400 μL DMEM respectively and stay for 5 min, then the PEI solution and plasmid solution were mixed and stay for 15 min, after that, the mixture was added to one 10 cm dish.

### 2.3 Proteins cross-linking

*In vitro* cross-linking reaction: In a 20 μL reaction, 10 μM Trx1 (in Hepes buffer, pH 8.0) and 10 μM thiol peroxidase (Tpx) or 3'-phosphoadenosine 5'-phosphosulfate reductase (PAPR) (in Hepes buffer, pH 8.0) were cross-linked at RT for 4 h with 0.5 mM BVSB/PDES, which corresponded to a 1:50 protein/cross-linker molar ratio. The cross-linking reaction was terminated at RT by adding 100 mM DTT (Sangon) and incubating for 20 min.

*E. coli* live cells cross-linking: Cell pellets from 50 mL cell culture were washed 3 times with PBS, resuspended with 400 μL PBS (final volume about 500 μL) and incubated with 2.5 mM BVSB or PDES (co-expression plasmids and 2 KR mutant plasmids) for 3 h (1 h spinning slightly at 4°C for cross-linkers entering cell, 2 h stand at RT for cross-linking). Then sample was centrifuged (5000 × g, 4°C, 1 min) to remove the upper liquid, followed by washed 3 times with PBS to remove the extra linkers thoroughly.

Mammalian live cells cross-linking: After 48 h, post-transfection 293T cells were scratched and washed 3 times with PBS. Cell pellets were resuspended with 800 μL PBS finally (final volume about 1.2 mL) and treated with 0.5 mM BVSB/PDES for 3 h (1 h spinning slightly at 4°C for linkers penetrating to cells，2 h stand at RT for cross-linking). Then sample was centrifuged (500 × g, 4°C, 1 min) to remove the upper liquid, followed by washing 3 times with PBS to remove the extra linkers thoroughly.

### 2.4 His-tag protein purification

*E. coli* cell pellets were resuspended in 12 mL lysis buffer (50 mM Tris-HCl pH 8.0, 500 mM NaCl, 20 mM imidazole, 1% v/v Tween 20, protease inhibitor cocktail), and mammalian cell pellets were lysed with 8 M urea buffer supplemented with protease inhibitor. Cell lysates were sonicated with Sonic Dismembrator (Fisher Scientific, φ6, 30% output, 10 min, 2 s off, 1 s on for *E. coli* cell lysates; φ3, 30% output, 5 min, 2 s off, 1 s on for mammalian cell lysates) in an ice-water bath, followed by centrifugation (21000 × *g*, 30 min, 4°C). The soluble fractions were collected and incubated with pre-equilibrated Ni-NTA Agarose resin (200 μL/100 mL culture, smart-lifesciences) at 4°C for 1 h with constant mechanical rotation. The slurry was loaded onto a Poly-Prep Chromatography Column (Sangon Biotech), washed with 5 mL of wash buffer 1 (50 mM Tris-HCl pH 8.0, 500 mM NaCl and 20 mM imidazole) for three times, and eluted with 200 μL of elution buffer (50 mM Tris-HCl, pH 8.0, 500 mM NaCl and 250 mM imidazole) for five times. The eluates were concentrated and buffer exchanged into protein storage buffer 1 (50 mM Hepes, pH 8.0, and 250 mM NaCl) using Amicon Ultra columns (Millipore), and stored at −80°C for future analysis.

### 2.5 Protein digestion and desalting

Protein samples were precipitated by six volumes of acetone at −20°C for 30 min. Precipitated proteins were dried in air and resuspended in 8 M urea, 100 mM Tris, pH 8.5. After reduction with 5 mM TCEP (Sigma) for 20 min and alkylation with 10 mM iodoacetamide (Sigma) for 15 min in the dark, samples were diluted to 2 M urea with 100 mM Tris, pH 8.5, and digested with trypsin (from Promega, at 50:1 protein: enzyme ratio) at 37°C for 16 h. Digestion was stopped by adding formic acid to 5% final concentration, and digested peptides were desalted with stage-tip. Trx1/PAPR and Trx1/Tpx *in vitro* cross-linking samples were further digested with Glu-C enzyme (from Promega, at 100:1 protein: enzyme ratio) at 37°C for 8–12 h, and desalted with stage-tip again.

### 2.6 Cross-linked peptides enrichment

Two-step purification of cross-linked peptides: Trx1 cross-linked complexes were purified as above His-tag purification. A total of 200 μg purified proteins were digested with Lys-C, and then digested peptides were diluted to 1 mL with incubation buffer (50 mM Tris, pH 8.0, 100 mM NaCl) and incubated with 40 μL Ni-NTA beads for 2 h. The beads were rinsed with wash buffer 1 for 3 times and incubation buffer for 2 times. On-beads digestion with Glu-C at 37°C for 8 h, and extract digested peptides with 50 μL incubation buffer for 3 times and desalting with stage-tip.

Strong cation exchange (SCX) enrichment: Trypsin digested peptides were loaded on 250 μm inter diameter column, which contains downstream 3 cm long reverse phase section (3 μm, C18 resin from Phenomenex), upstream 4 cm long SCX phase section (5 μm, resin from Whatman) and a frit at the end. Desalt with Buffer A (0.1% FA) and peptides were eluted from reverse phase to SCX phase with Buffer B (100% ACN, 0.1% FA). 12 fractions were collected from a series of concentration gradients of ammonium acetate (each was 5 μL: 75 mM, 100 mM, 150 mM, 275 mM, 375 mM, 500 mM, 650 mM, 800 mM, 900 mM, and 3 times of 1 M). These fractions were desalted, and 7 fractions (from 500 mM to 1 M_3) of 293T sample were analyzed by mass spectrometry.

### 2.7 Biotin switch assay

Cell lysates or purified proteins were incubated with the S-methyl methanethiosulfonate (MMTS, Sigma-Aldrich) buffer (10 mM MMTS, 2.5% SDS, 100 mM Hepes, pH 7.4, 1 mM EDTA, 0.1 mM neocuproine) to block free thiol groups, and S-nitrosothiols of proteins were reduced by 20 mM ascorbate. The newly generated free thiols were biotinylated with 1 mM biotin-HPDP (APExBIO Technology). For control samples, ascorbate was omitted from the procedure. Biotinylated proteins were purified with NeutrAvidin agarose (Thermo Fisher Scientific), and the purified proteins were subjected to immunoblot analysis.

### 2.8 Transnitrosylation assay

His-tag purified TXN1 was treated with decayed (old) or freshly prepared (new) 100 mM SNOC in lysis buffer (PBS containing 0.1% Triton X-100) for 30 min at room temperature in the dark with gentle shaking. Concurrently, HEK293T cells expressing CASP6-FLAG were lysed in lysis buffer with sonication. Old or new SNOC treated TXN1s were added to HEK293T cell lysates and incubated for 1 h at room temperature in the dark with gentle shaking, respectively. The reaction mixtures were subsequently subjected to biotin switch assay after addition of MMTS blocking buffer.

### 2.9 S-octyl 2-(diphenylphosphino) benzothioate (SDBP) treatment of Flag-PLK1

Purified Flag-PLK1 was mixed with 100 μL labeling buffer (1 mM SDBP, THF:PBS 3:1) at room temperature for 4 h with gentle shaking. Proteins were precipitated by six volumes of acetone at −20 °C for 30 min.

### 2.10 Mass spectrometry analysis

Peptides were loaded on analytical column (75 × 15 cm, 1.9 μm C18, 1 μm tip) with Easy-nLC 1200 system. Samples were analyzed with a 60 min gradient at flow rate 300 nL min−1 as follows: 2–8% B for 2 min, 8–27% B for 43 min, 27–35% B for 8 min, 35–100% B for 3 min, and 100% B for 4 min. Q Exactive HF-X mass spectrometer was operated in data-dependent mode with one full MS scan at R = 60 000 (*m*/*z* 200), followed by 20 HCD MS/MS scans at R = 15 000, NCE = 27, with an isolation width of 1.6 *m*/*z*. The AGC targets for MS1 and MS2 scans were 1 × 106 and 5 × 104, respectively, and the maximum injection time for MS1 and MS2 were 20 and 45 ms, respectively. Precursors of +1, +8, or above, or unassigned charge states were rejected; exclusion of isotopes was disabled; dynamic exclusion was set to 45 s.

### 2.11 Data analysis

Cross-linked peptides were identified using pLink2 software [23]. pLink search parameters: precursor mass tolerance 20 parts per million (ppm); fragment mass tolerance 20 ppm; filter tolerance 10 ppm; False Discovery Rate ≤ 5 at peptide spectrum match level; peptide length minimum 6 amino acids and maximum 60 amino acids per chain; peptide mass minimum 600 and maximum 6,000 Da per chain, variable modification Cys 57.02146; enzyme: Trypsin and Glu-C for *in vitro* cross-linking samples, Lys-C and Glu-C for *in vivo* samples of *E. coli*, and trypsin for 293T samples; three missed cleavage sites per chain. Protein sequences were downloaded from Uniprot. BVSB was defined as 258.00150 Da and PDES was defined as 288.02330 Da in pLink2.

Gene ontology (GO) term and KEGG pathway enrichment for functional analysis were performed using the clusterProfiler package under the R software [24]. *E. coli* proteome or human proteome were used as the background for enrichment analysis. Significance of enrichment analysis was defined using hypergeometric test, and the p values were corrected for multiple hypothesis testing using the BH method [25]. Enriched terms and pathways were filtered according to the adjusted p values.

## 3 Results

**Fig. 1:**
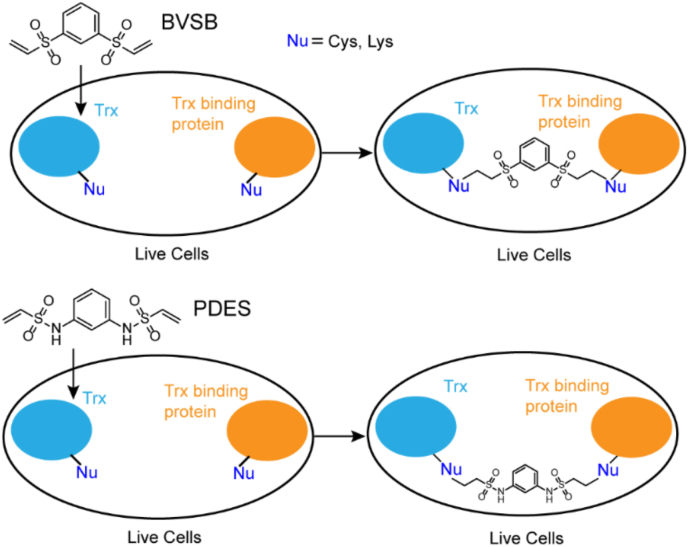
Cell permeable cross-linkers BVSB and PDES for live cells cross-linking.

The binding interfaces of oxidoreductases and their substrates contain cysteine residues. Previously, a short homobifunctional cross-linker DVSF with vinyl sulfone groups was designed to target these cysteine residues at the binding interfaces [[20], [21], [22]]. However, this cross-linker doesn’t allow further derivatization to install chemical handles, such as alkyne and biotin groups for enrichment or other groups to tune the reactivity of vinyl sulfone. Therefore, we designed and synthesized two cross-linkers with bis-vinyl sulfones bridged by rigid linkers (Fig. 1, Figs. S1–6), BVSB (8.7 Å) and PDES (11.8 Å). Both cross-linkers are expected to be cell permeable because of their small size and neutral charge. In protein cross-linking involving oxidoreductases, the vinyl sulfone groups of BVSB and PDES will primarily react with side chains of Cys to covalently fix transient protein-protein interactions. After purification and enrichment, targeted protein complexes will be digested with proteases and analyzed by mass spectrometry to identify inter-protein cross-linked peptides. These cross-linked peptides will inform which proteins directly bind to target proteins.

### 3.1 *In vitro* cross-linking of Trx1 and its interacting proteins with BVSB and PDES

**Fig. 2:**
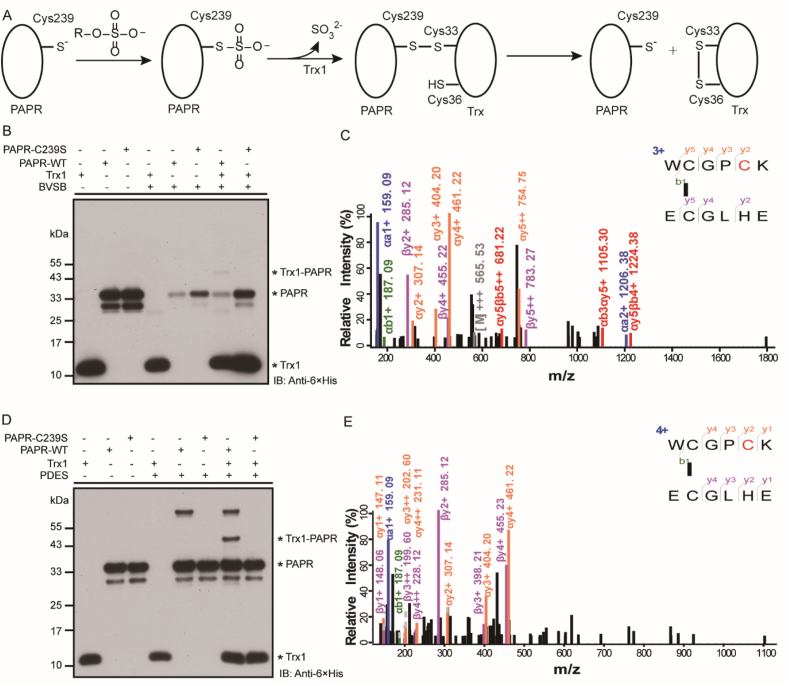
*In vitro* cross-linking of Trx1 and its substrates. A) Mechanism of reducing PAPR by Trx1. B) Western blot analysis of Trx1 cross-linked with PAPR or the PAPR C239S mutant using BVSB. C) Tandem mass spectrum showing BVSB cross-linking between Trx1 and PAPR active sites. D) Western blot analysis of Trx1 cross-linked with PAPR or the PAPR C239S mutant using PDES. E) Tandem mass spectrum showing PDES cross-linking between Trx1 active site and PAPR active site.

First, we evaluated BVSB cross-linking of Trx1 with one of its known protein binders PAPR *in vitro*. PAPR catalyzes the formation of sulfite from 3'-phosphoadenosine 5'-phosphosulfate with a mechanism involving binding to Trx1 to restore the active enzyme (Fig. 2A) [26,27]. To covalently capture the PAPR/Trx1 complex, His-tagged Trx1 and PAPR were incubated with BVSB or PDES for 4 h at room temperature (Figs. S7–8). As shown in Fig. 2B and 2D, cross-linking bands corresponding to a covalent complex of the Trx1/PAPR heterodimer were detected on Western blot. To further confirm this inter-protein cross-linking and map cross-linking sites, cross-linked protein samples were digested by Trypsin and Glu-C before analysis by mass spectrometry. Interestingly, only inter-protein cross-linked peptides bridging Trx1 (33) and PAPR (239) were identified (Fig. 2C and 2E). The cross-linking sites are cysteine residues corresponding to enzymatic active sites of Trx1 and PAPR, which are close to each other in the crystal structure (Fig. S9). When the active-site Cys239 was mutated to Ser, no cross-linking band was detected (Fig. 2B and 2D), highlighting the specificity of BVSB and PDES cross-linking to target active-site cysteine residues in close proximity. Importantly, although Trx1 binds to the reduced form of PAPR with a relatively low binding affinity (K_D_ ∼110 μM) [27], BVSB and PDES still successfully captured this weak protein-protein interaction.

We tested BVSB and PDES on another protein pair, Trx1 and Tpx. Tpx catalyzes the reduction of peroxides and protects cells against oxidative stress [28]. After incubation of BVSB with Trx1/Tpx, a cross-linking band corresponding to the cross-linked Trx1/Tpx complex was observed on Western blot (Fig. S10A). Through MS analysis, an inter-protein cross-linked peptide between two active sites Tpx (61)-Trx1 (33) was detected (Fig. S10B), while the double mutant of Tpx (C61S and C95S) showed no cross-linking (Fig. S10A). Similar results were obtained when applying a longer linker PDES (Fig. S10 C-D). Taken together, *in vitro* cross-linking of Trx1 and its binders with vinyl sulfone based cross-linkers successfully captured heterodimers by linking active-site cysteine residues, while inactivation of enzymes by Cys-to-Ser mutation diminished cross-linking.

### 3.2 *In vivo* cross-linking of Trx1 and its interacting proteins with BVSB and PDES

**Fig. 3:**
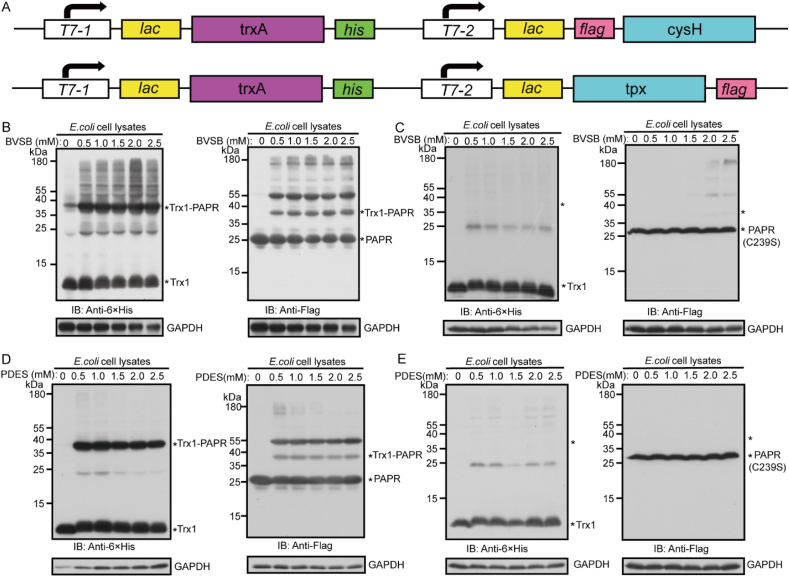
Cross-linking of Trx1 and its substrates in live *E. coli* cells. A) Constructs that co-expressing Trx1/PAPR and Trx1/Tpx complexes. B-E) Western blot analysis showing successful cross-linking of Trx1 and its substrate PAPR in live cells by BVSB (B) or PDES (D). No cross-linking band detected for Trx1 and PAPR C239S mutant after treating live cells with BVSB (C) or PDES (E).

With encouraging *in vitro* results, we further tested BVSB cross-linking in live *E. coli* cells by co-expressing His-tagged Trx1 and Flag-tagged PAPR (Fig. 3A). Live cells were treated with different concentrations of BVSB before quenching and cell lysis. Subsequent Western blot analysis under denaturing conditions identified a cross-linked band of Trx1 and PAPR heterodimer visible using both anti-His and anti-flag antibodies (Fig. 3B). However, when Cys239 of PAPR was mutated to Ser, the Trx1/PARP cross-linking was eliminated (Fig. 3C). With MS analysis, an inter-protein cross-linked peptide bridging Trx1 (33)-PAPR (239) was successfully identified (Fig. S11A). Similar results were obtained when using the PEDS cross-linker (Fig. 3D–E, Fig. S11B). His-tagged Trx1 and Flag-tagged Tpx were also successfully cross-linked by BVSB and PDES in live cells (Figs. S12–13). These results demonstrated that both BVSB and PEDS are cell-permeable cross-linkers, and they are excellent at targeting Cys residues at the binding interface of Trx1 and its interacting proteins.

### 3.3 Identification of direct binding proteins of Trx1 in *E. coli* with BSVB and PDES

**Fig. 4:**
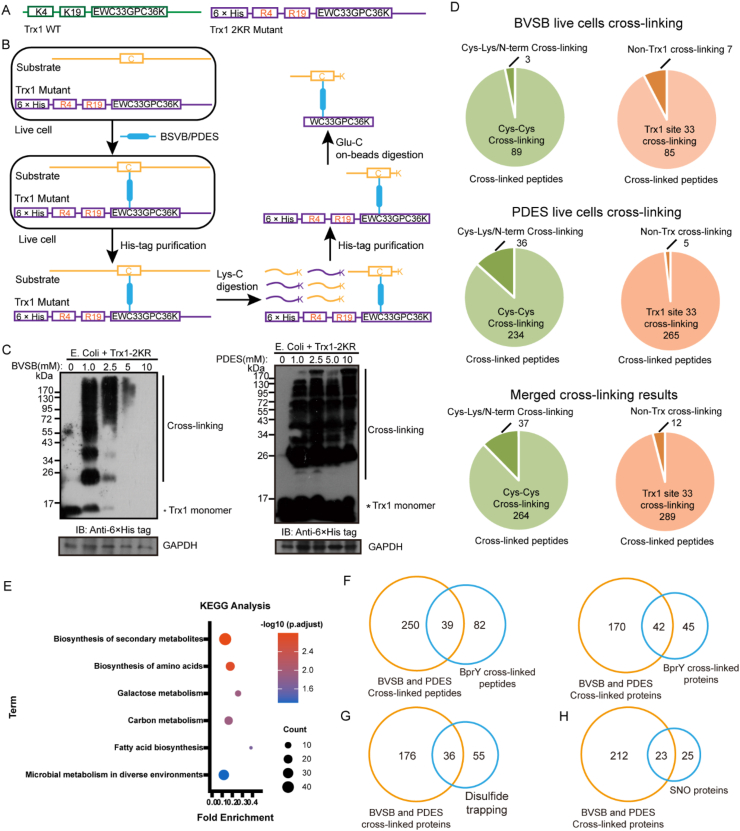
Identify direct interactome of Trx1 in live *E. coli* cells using chemical cross-linking. A) Constructs of Trx1 WT and Trx1* (Trx1-2KR) for live cell cross-linking. This mutant enables the two-step purification step to enrich cross-linked peptides. B) Workflow of a two-step purification to enrich cross-linked peptides for MS analysis. C) Western blot analysis of cell lysates obtained from BVSB or PDES treated *E. coli* cells, showing multiple endogenous proteins cross-linked to Trx1. D) Types of cross-linked peptides from BVSB or PDES cross-linked Trx1 complexes. E) KEGG analysis of Trx1 interacting proteins from this study. Comparison of identified cross-linked peptides and interacting proteins from this study with those identified using an unnatural amino acid BprY (F) [29], disulfide trapping(G) [5], and SNO (H) [30].

To explore the ability of BVSB and PDES to capture Trx1 interacting proteins in live cells, we constructed a His-tagged Trx1 construct containing K4R and K19R mutations (Trx1*, Fig. 4A). These mutations have no effect on the protein function but make it compatible with a two-step purification method to enrich cross-linked peptides (Fig. 4B) [12]. Live *E. coli* cells expressing Trx1* were treated with BVSB or PDES to fix protein interactions between Trx1* and its binding proteins. After cell lysis, cross-linked Trx1* protein complexes were immobilized onto Ni-NTA beads followed by digestion with Lys-C. Because there is no lysine residue between His-tag and Cys33 in Trx1*, it remains intact and bound to Ni-NTA after Lys-C treatment together with cross-linked peptide segments from interacting proteins. A second round on-beads Glu-C digestion releases cross-linked peptides for MS analysis (Fig. 4B). This two-step purification method effectively reduced the complexity of MS sample analysis to help identify more cross-linked peptides. After live cell cross-linking, intense higher MW bands were visible on the Western blot, indicating successful cross-linking of Trx1* to its interacting proteins in live cells by both BVSB and PDES (Fig. 4C).

Enriched cross-linked peptides were subject to MS analysis. A total of 92 cross-linked peptides were identified for BVSB and 270 identified for PDES in all 3 MS technical replicates (Fig. 4D and Tables S1–3). After combining results from BVSB and PDES cross-linking to remove redundant peptides, we identified a total of 301 cross-linked peptides. The majority (264/301) of them are Cys-Cys cross-linked peptides, and 289 out of 301 cross-linked peptides involve active-site Cys33 of Trx1* as the targeted residue, corresponding to 285 inter-protein cross-links. To the best of our knowledge, the largest dataset prior to this work was the identification of 176 inter-protein cross-links from 14 proteins IP experiments and on-beads cross-linking [19]. Here, we identified more than 200 inter-protein cross-links from a single protein pull-down experiment and live cell cross-linking, which demonstrated that our two-step purification strategy can effectively enrich cross-linked peptides involving Trx1. This result demonstrated that BVSB and PDES were able to effectively cross-link enzyme active sites with their binding proteins. This analysis led to the discovery of 212 putative protein binders of Trx1. KEGG enrichment analysis showed that these proteins are involved in multiple metabolism pathways (Fig. 4E), consistent with the important role of Trx1 in regulating metabolism. We also identified cross-links between Trx1 Cys33 with active sites of other enzymes, including DAPDC, GatD, AceA, FabB, FabH, and PpsA (Fig. S14). These cross-links indicate that Trx1 Cys33 is close to active sites of the above enzymes, suggesting Trx1 might be involved in maintaining activities of these enzymes [5].

Previously, we identified 121 cross-linked peptides by incorporating genetically encoded unnatural amino acid BprY onto residue 33 of Trx1 [29], leading to the discovery of 87 interacting proteins. The study here covered ∼32% BprY cross-linked peptides and ∼48% BprY cross-linked proteins (Fig. 4F), indicating that these two approaches are complementary to each other. Through comparison with other studies, we identified 36 of 91 Trx1 substrates of disulfide trapping strategy (Fig. 4G) [5] and 23 of 48 previously published SNO proteins in *E. coli* (Fig. 4H) [30].

### 3.4 Identification of SNO substrates of thioredoxin 1 (TXN1) in HEK293T cells with BVSB and PDES

**Fig. 5:**
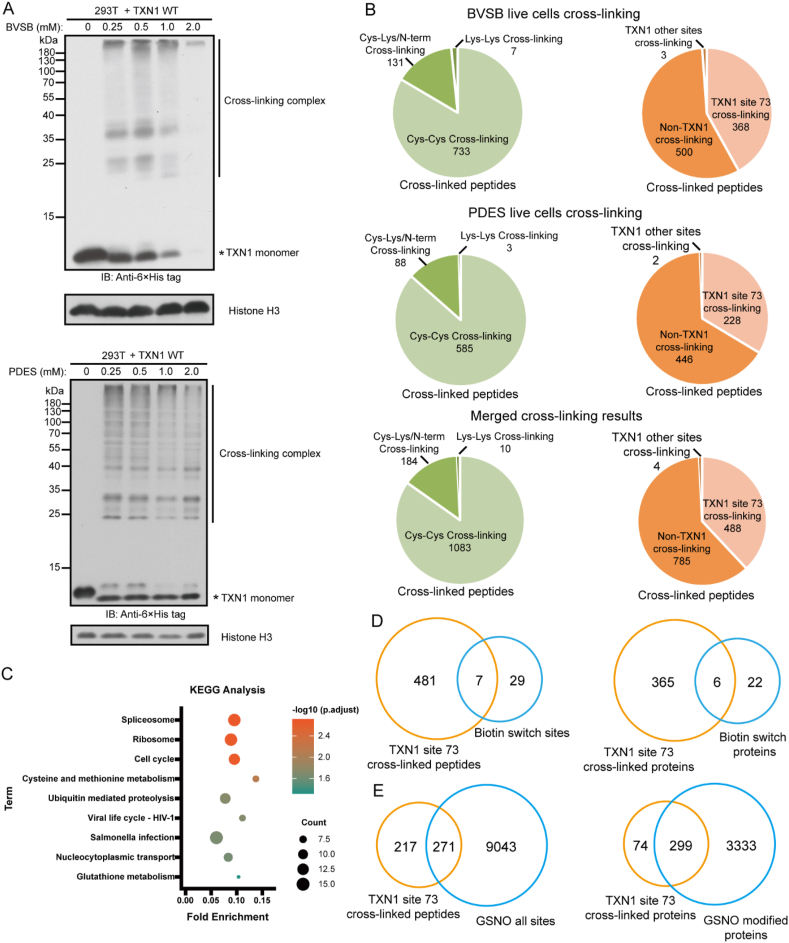
Identify the SNO substrates of TXN1 in live HEK293T cells. A) Western blot analysis of cell lysates from BVSB or PDES treated 293T cells, showing multiple endogenous proteins cross-linked to TXN1. B) Types of cross-linking peptides from BVSB and PDES cross-linked TXN1 complexes. C) KEGG analysis of TXN1 interacting proteins from this study. D-E) Venn diagram showing the overlap of SNO substrates from this study with previously identified SNO modified sites using a global proteomics approach (D) and a chemical proteomics strategy (E).

In mammalian cells, TXN1 is involved in not only disulfide exchange reactions, but also reversible SNO of cysteine residues [31]. A previous chemical proteomics study has identified 3,632 potential SNO-targeted proteins in cell extracts after the treatment with S-nitrosoglutathione (GSNO), showing the ubiquity of SNO modification [32]. While Cys32 and 35 residues in TXN1 are important to disulfide exchange reactions, Cys73 is believed to catalyze the transnitrosylation [[31], [33]]. Therefore, we hypothesize that within 3,632 potential SNO-targeted proteins, those that can be cross-linked with TXN1 at Cys73 are putative SNO substrates of TXN1. To test this hypothesis, we transfected HEK293T cells with a plasmid expressing His-tagged TXN1 and performed live cell cross-linking using BVSB or PDES. Western blot analysis showed higher MW bands after cross-linking with BVSB or PDES (Fig. 5A), suggesting successful capture of TXN1 binders. After Ni-NTA purification and trypsin digestion, the peptide mixture was fractionated using an off-line SCX column. MS analysis identified 871 cross-linked peptides for BVSB cross-linking and 676 for PDES cross-linking (Fig. 5B and Tables S4–6). In total, we identified 1277 cross-linked peptides among which 488 were cross-linked at TXN1 Cys73, corresponding to 373 leading proteins. KEGG analysis showed that they are involved in spliceosome, ribosome, cell cycle, *etc*. (Fig. 5C). Our data confirmed 7 published SNO modified peptides and 6 SNO protein substrates of TXN1 (Fig. 5D) [33]. Compared with the 8,304 identified SNO sites in S-nitrosoglutathione (GSNO) treated HeLa cell lysates [32], our results confirmed 271 sites (Fig. 5E), suggesting these sites could be co-regulated by both GSNO and TXN1. The majority (299 out of 373) of proteins were within 3,632 potential SNO-targeted proteins. We believe these 299 proteins are high-confidence SNO substrates of TXN1. It is well known that Thioredoxin Reductase 1 (TXNRD1) Cys647-Sec648 catalyzes the reduction of TXN1 Cys32-Cys35 disulfide bond. Interestingly, we also identified TXNRD1 Sec648 – TXN1 Cys73 cross-linked peptide (Fig. S15A). Since TXNRD1 Sec648 is also close to TXN1 Cys73 (Fig. S15B), TXNRD1 could also reduce TXN1 Cys73.

**Fig. 6:**
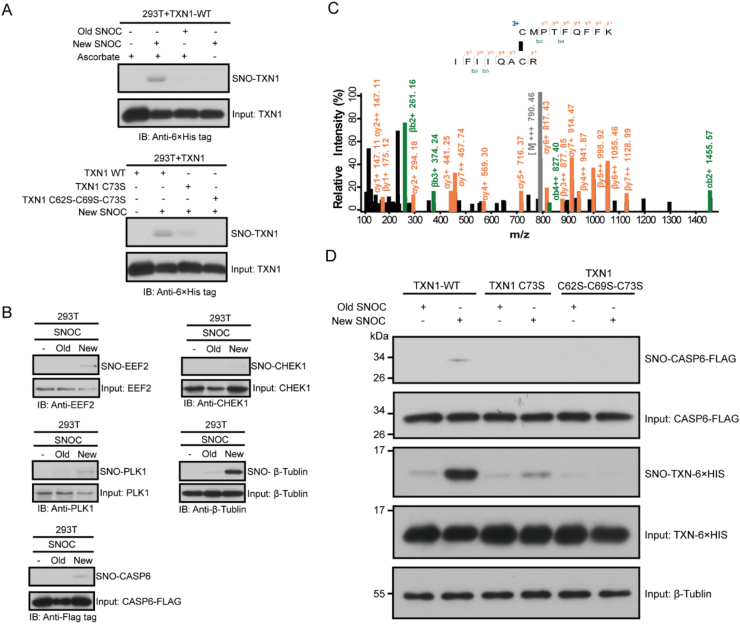
Validation of SNO modified proteins using a biotin switch assay. A) Cys73 of TXN1 was the main SNO site of TXN1 as validated by the biotin switch assay. (“Old” SNOC represents the negative control with decayed SNOC, and “new” SNOC is freshly prepared SNOC). B) Biotin switch assays confirmed SNO-modification on EEF2, CHEK1, PLK1, β-tublin, and Caspase 6. C) Tandem mass spectrum supporting a cross-link between TXN1(73)-CASP6(163). D) TXN1 catalyzed transnitrosylation on caspase 6.

To validate our approach, we setup biotin switch assay to detect SNO proteins [34], this method was confirmed by *in vitro* SNO assays of TXN1 and its mutants (Fig. 6A). We randomly chose three proteins EEF2, CHEK1 and PLK1 within our protein list and carried out biotin switch assays to analyze the extent of SNO by S-nitrosocysteine (SNOC). Western blot analyses demonstrated that these proteins can be S-nitrosylated *in vitro* (Fig. 6B). In addition to the biotin switch assay, we also synthesized a phosphine-based probe S-octyl 2-(diphenylphosphaneyl) benzothioate (SDBP, Figs. S16–S18). This probe can selectively react with SNO of proteins to form a disulfide bond [35,36]. Using this probe, we identified an SNO site of PLK1, confirming our result from the biotin switch assay (Fig. S19). β-tublin as a S-nitrosylated candidate protein has been identified in a published study and our dataset [37], but not in GSNO treated cells. We confirmed that β-tublin can be S-nitrosylated *in vitro* (Fig. 6B). A cross-link between Caspase 6 Cys163 and TXN1 Cys73 was identified by searching against a small protein database (Fig. 6C). We show that Caspase 6 can be S-nitrosylated by SNOC *in vitro* and this modification is catalyzed by TXN1 dependent on Cys73 (Fig. 6D). Interestingly, SNO of Caspase 6 at Cys146 has been reported in nitrite treated hypoxic neuro-2a cells [38]. Our result suggests that TXN1 could be an important player in this process.

### 3.5 Expand chemical cross-linking to capture direct binding proteins of other oxidoreductase

**Fig. 7:**
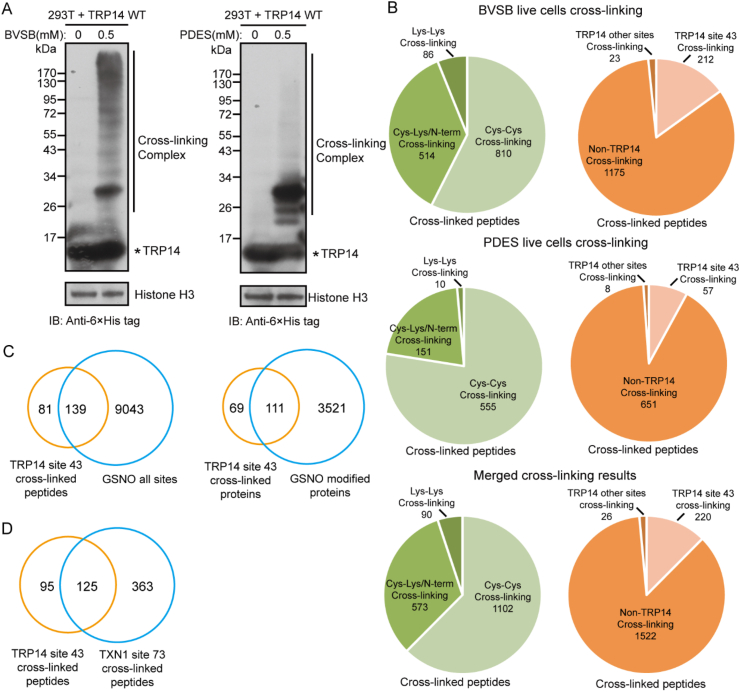
Identify direct interactome of TRP14 in live HEK293T cells. A) Western blot analysis of cell lysates from BVSB or PDES treated 293T cells, showing multiple endogenous proteins cross-linked to TRP14. B) Types of cross-linked peptides from BVSB and PDES cross-linked TRP14 complexes. C) Venn diagrams showing overlap of identified cross-linked peptides from this study with published SNO modified sites. D）Cross-linked peptides identified by TRP14 and TXN1 cross-linking.

To demonstrate the versatility of our approach, we applied live cell cross-linking to study Thioredoxin related protein 14 (TRP14), a member of Trx-fold protein family, also showing reductase and denitrosylation activities [39]. HEK293T cells expressing His-tagged TRP14 were treated with BVSB and PDES, separately. Western blot analysis showed TRP14 was cross-linked with its binding proteins by BVSB or PDES (Fig. 7A). After MS analysis, a total of 1768 cross-linked peptides were identified, among which 220 cross-linked peptides contained TRP14 Cys43 as one of the cross-linking sites, leading to the discovery of 180 protein binders of TRP14 (Fig. 7B and Tables S7–9). These peptides confirmed 139 SNO sites from GSNO treated cell lysates and 111 SNO proteins (Fig. 7C). A total of 125 peptides were cross-linked by active sites of TRP14 and TXN1 (Fig. 7D), potentially proteins corresponding to these peptides are co-regulated by TRP14 and TXN1. This study demonstrated that our cross-linking strategy is highly robust in the interactome analysis of oxidoreductases.

## 4 Discussion

Protein cross-linking is a two-step process. In the first step, one reactive end group of a cross-linker reacts with an amino acid sidechain on the protein surface. Then, the other reactive end group reacts with another amino acid sidechain to form intra- or inter-protein cross-linking. The second step is a first-order reaction for intra-protein cross-linking but a second-order reaction for inter-protein cross-linking. Therefore, different from intra-protein cross-linking, the reaction rate of inter-protein cross-linking depends on the concentrations of two interacting proteins. Because the concentrations of endogenous proteins are low, it is more difficult to form inter-protein cross-linking than intra-protein cross-linking. This is supported by results from a previous study in which an average of ∼12 inter-protein cross-links were identified from one affinity purification and on-beads cross-linking experiment [19].

The abundance of cysteine residues in proteins is relatively low, and many of them are located in hydrophobic regions of proteins or in the form of disulfide bonds [40,41]. Therefore, the cross-linkable cysteine residues on the protein surface are limited. When using a cysteine-targeting cross-linker in comparison with more commonly used lysine-targeting cross-linkers, the probability of forming intra-protein cross-linking should be decreased, and the possibility of inter-protein cross-linking should be increased. Active-site cysteine of oxidoreductase has close contact with cysteine residues of its substrates, so it is convenient to generate inter-protein cross-links between substrates and oxidoreductase using cysteine-targeting cross-linkers. This aspect has been demonstrated by results in this study, supporting that cysteine reactive cross-linkers are powerful molecules to capture weak and transient interactions of oxidoreductase and its substrates to form inter-protein cross-linking.

The biotin switch method has been widely used to detect SNO modifications, but it is a challenging and labor-intensive *in vitro* assay using cell lysates [42]. Subcellular organelles have different redox states. For example, mitochondria and nuclei have reduced redox potential, while cytoplasm contains metabolic oxidases. Secretory pathways have oxidative systems for disulfide bond formation [43]. Therefore, the analysis of cell lysates with disrupted subcellular compartments could lead to false positive results. We believe it is important to capture substrates of oxidoreductases in live cells. We have demonstrated that cysteine-selective cell-permeable cross-linkers BVSB and PDES can site-specifically fix the interactions of oxidoreductase and their substrates in the most native environment.

The limitation of our methodology is that BVSB and PDES can react with reduced thiol but not disulfide bonds nor SNO of proteins. However, based on the reduction mechanism of Trx1, the attack of Trx1 Cys33 on disulfide bond of the substrate yields a reaction intermediate in which free thiol groups exist at the binding interface of both Trx1 and its substrate. This reaction intermediate could be captured by BSVB and PDES. Our study on SNO is based on the hypothesis that within the 3,632 potential SNO-target proteins, those proteins that can be cross-linked with TXN1 at Cys73 are putative SNO substrates of TXN1 with higher confidence. Our methodology is helpful to identify direct interacting proteins of enzymes. Potentially, it can identify substrates of enzymes in large-scale, but these substrate candidates need further biological validation. Future work will involve the development of cross-linkers directly targeting SNO on proteins for mapping direct substrates of enzymes.

## 5 Conclusions

In conclusion, we have demonstrated that Cys-targeting cross-linkers, such as BVSB and PDES with vinyl sulfone groups, can capture weak and transient protein-protein interactions between oxidoreductases and their substrates not only *in vitro* but also in live cells. Applying live cell cross-linking using BVSB and PDES and a two-step enrichment strategy, we have identified 212 putative protein binders of Trx1 in *E. coli*. Additionally, we applied this strategy to identify 299 SNO substrates of TXN1 and 111 SNO substrates of TRP14 in HEK293T cells. This study opens a new avenue for identifying substrates of enzymes in native cellular environments using chemical cross-linking and mass spectrometry. We believe that the XL-MS strategy can also be applied to the identification of substrates of other types of enzymes, such as phosphatases, deubiquitinases, protease, *etc*, which will inspire further development of new cross-linking technologies.

## Author contributions

T.W., S.T.L., and Y.R. contributed equally to this work. T.W. and Y.R. designed and conducted experiments and data analysis. X.Z. performed mass spectrometry samples preparation. L.L. performed mass spectrometry samples measurements. S.T.L. and L.Z. performed bioinformatics analysis. Y.L. synthesized chemical cross-linkers. D.W. provided critical suggestions and contributed to writing. B.Y. and S.T. directed the project and wrote the manuscript.

## Declaration of competing interest

The authors declare no competing financial interest.

## Data Availability

Data will be made available on request.
