# Supplementary material for: Mapping protein direct interactome of oxidoreductases with small molecular chemical cross-linkers in live cells
Source: Redox Biol. 2023 Feb 24;61:102642. doi: 10.1016/j.redox.2023.102642 (PMC9986639; doi:10.1016/j.redox.2023.102642)
Supplement: Multimedia component 10 [file mmc10.docx]

Supporting Information

Mapping Direct Interacting Proteins of Oxidoreductases with Small Molecular Chemical Cross-linkers in Live Cells

Ting Wu, Shang-Tong Li, Yu Ran, Yinuo Lin, Lu Liu, Xiajun Zhang, Lianqi Zhou, Long Zhang, Donghai Wu, Haifan Wu, Bing Yang*, Shibing Tang*

**Table of Contents**

**1. Supplementary Figures**

Figure S1. Synthesis of the chemical cross-linker BVSB.

Figure S2. Synthesis of the chemical cross-linker PDES.

Figure S3. ^1^H NMR spectrum of BVSB.

Figure S4. ^13^C NMR spectrum of BVSB.

Figure S5. ^1^H NMR spectrum of PDES.

Figure S6. ^13^C NMR spectrum of PDES.

Figure S7. SDS-PAGE analysis of His-tag purified Trx1, PAPR and Tpx.

Figure S8. Optimization of BVSB cross-linking conditions.

Figure S9. Trx1 Cys33 and PAPR Cys 239 in close position on protein structure (PDB ID 2O8V).

Figure S10. Cross-linking of Trx1 and Tpx with BVSB and PDES.

Figure S11. Cross-linking of Trx1/PAPR in live cells.

Figure S12. Cross-linking of Trx1 and Tpx in live cells.

Figure S13. Cross-linking of Trx1/Tpx in live cells.

Figure S14. Mass spectra of Trx 1 cross-linked with active sites of DAPDC, GatD, AceA, FabB, FabH, PpsA.

Figure S15. BVSB cross-links TXNRD1 (Sec648) with TXN1 (Cys73).

Figure S16. Synthesis of SDBP.

Figure S17. ^1^H NMR spectrum of SDBP.

Figure S18. ^13^C NMR spectrum of SDBP

Figure S19. Validation of SNO site with phosphine compound.

**2. Supplementary Notes**

**1. Supplementary Figures**

**Figure S1**. Synthesis of the chemical cross-linker BVSB.

**Figure S2**. Synthesis of the chemical cross-linker PDES.

**
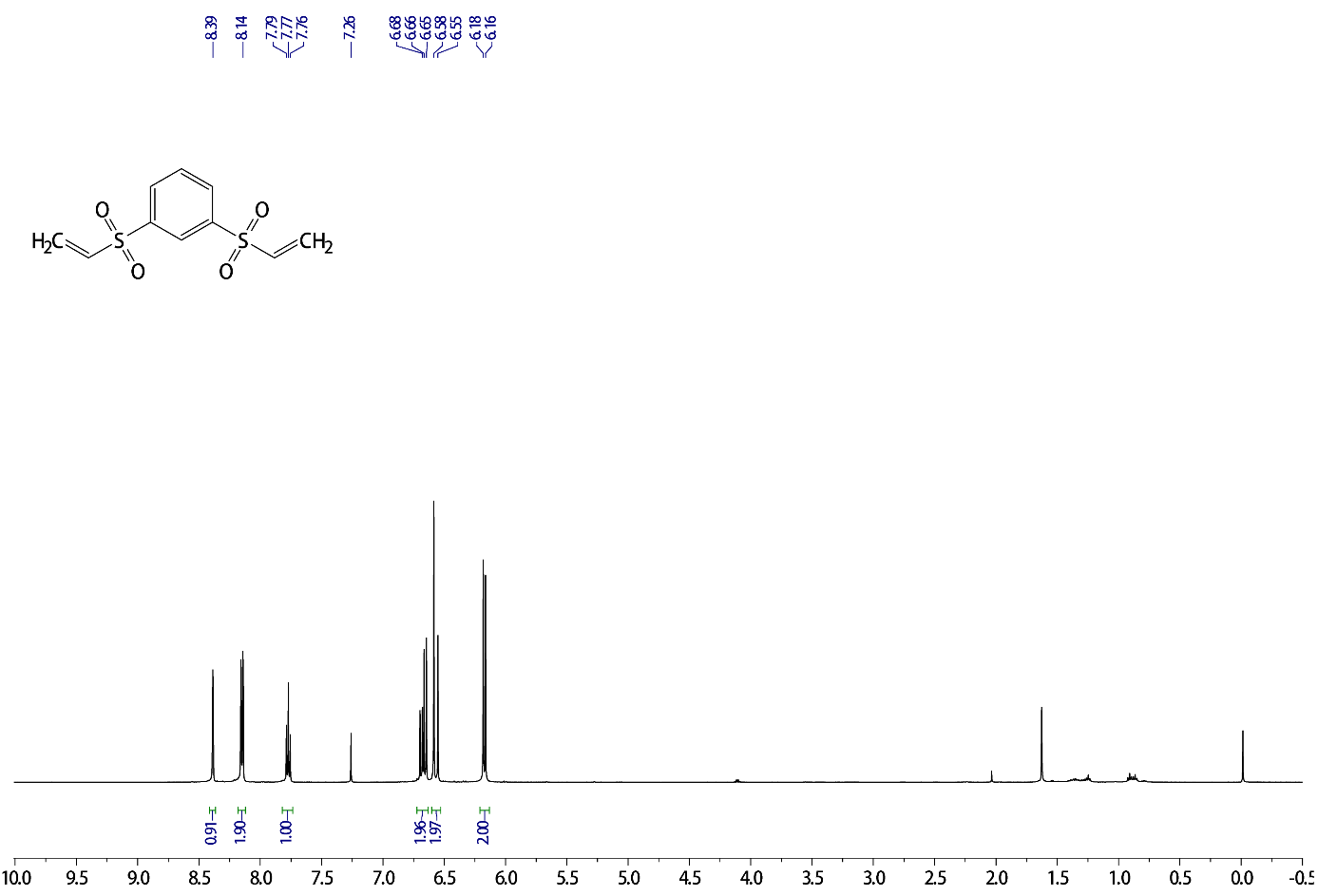
**

**Figure S3.** ^1^H NMR spectrum of BVSB.

**
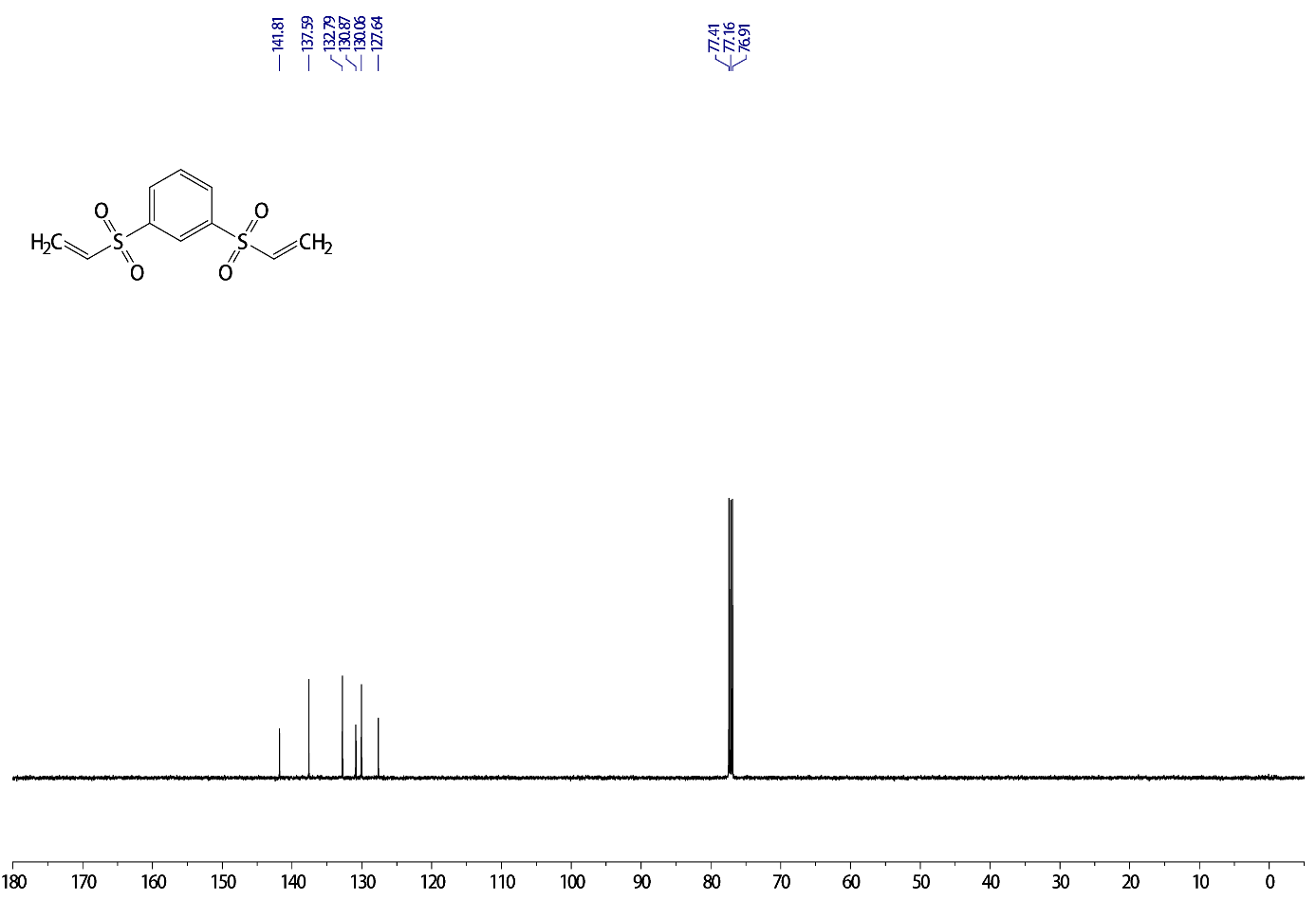
**

**Figure S4.** ^13^C NMR spectrum of BVSB.


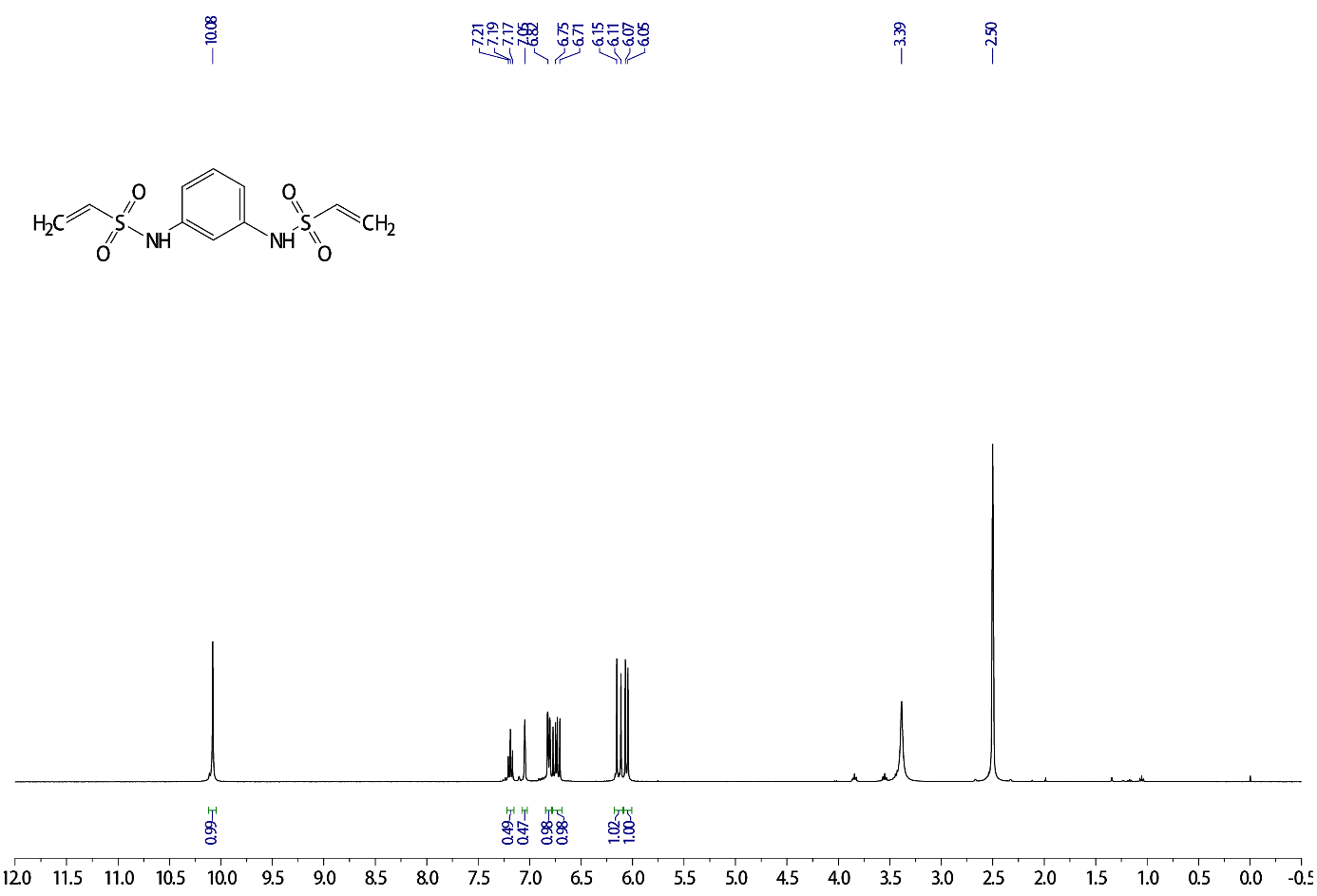


**Figure S5.** ^1^H NMR spectrum of PDES.


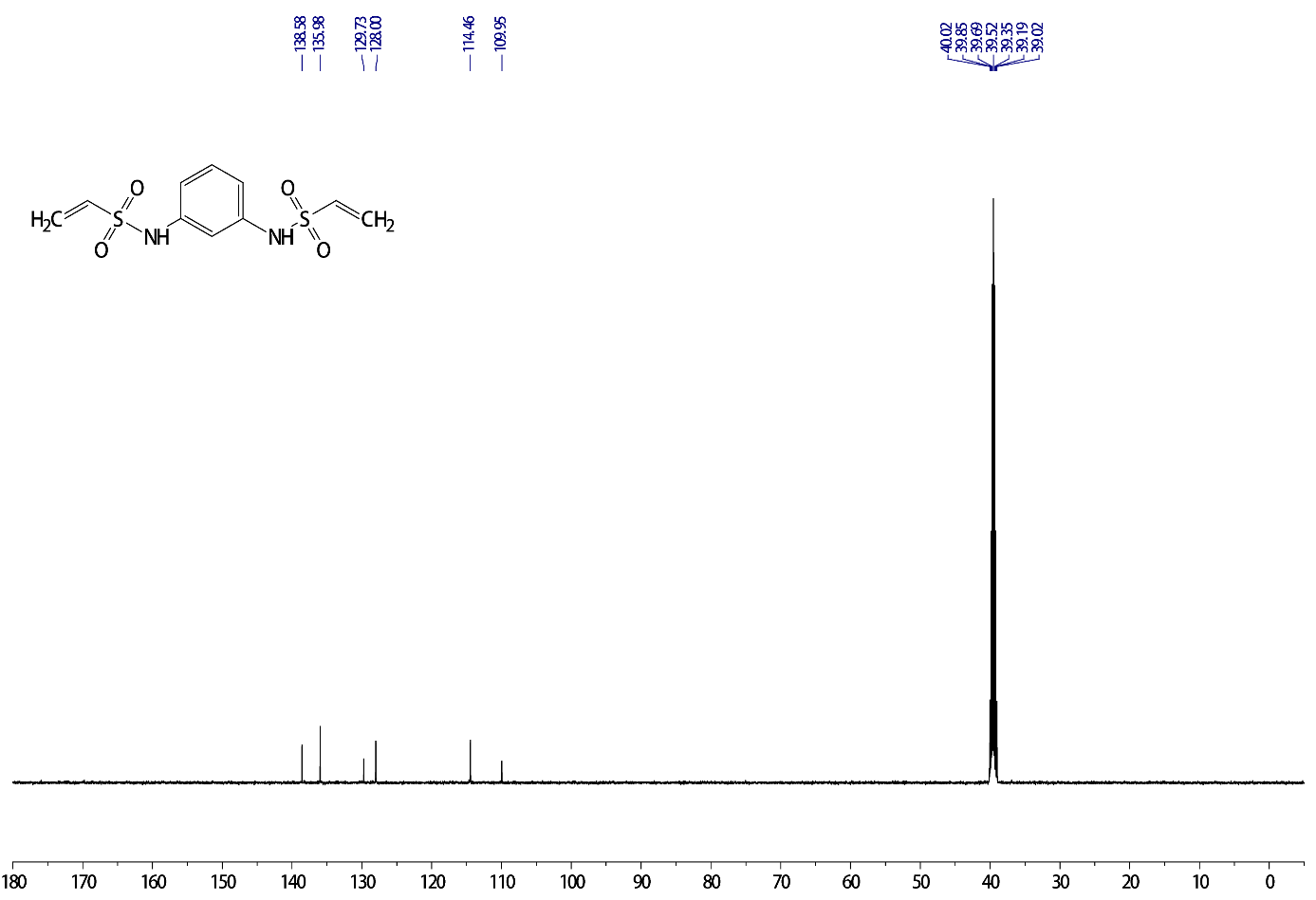


**Figure S6.** ^13^C NMR spectrum of PDES


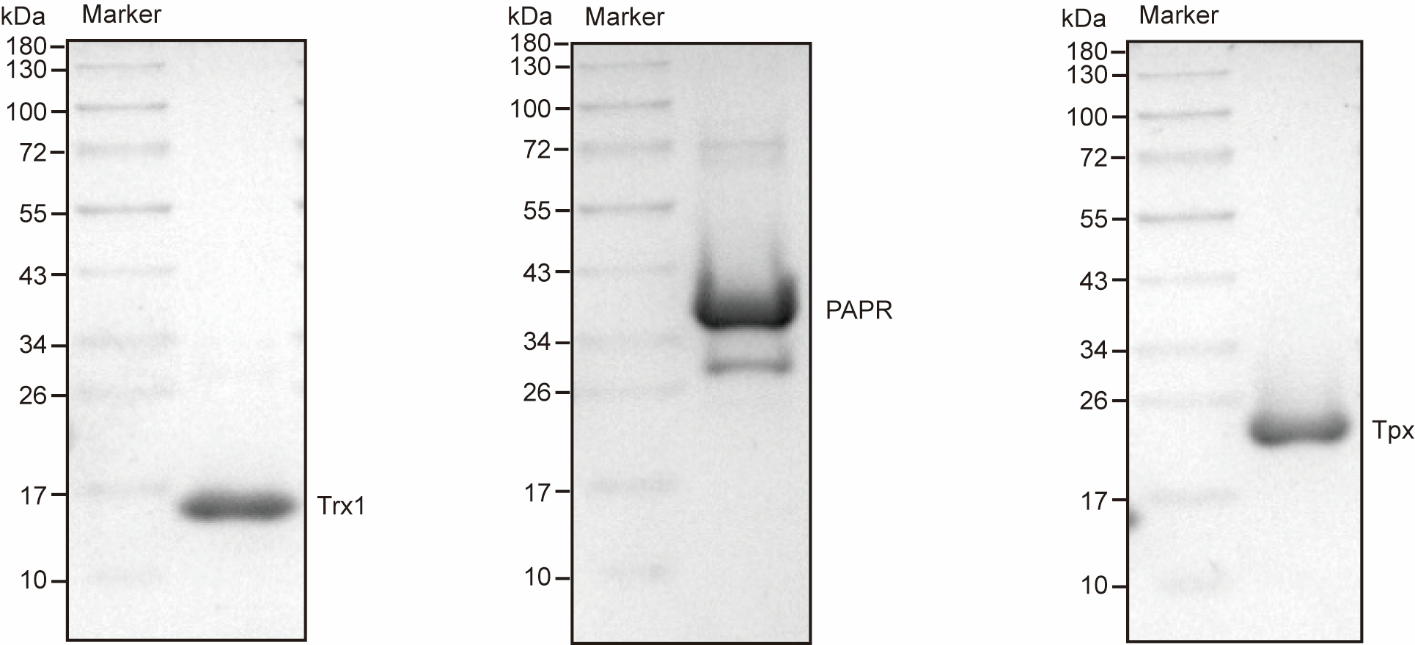


**Figure S7.** SDS-PAGE analysis of His-tag purified Trx1, PAPR and Tpx.


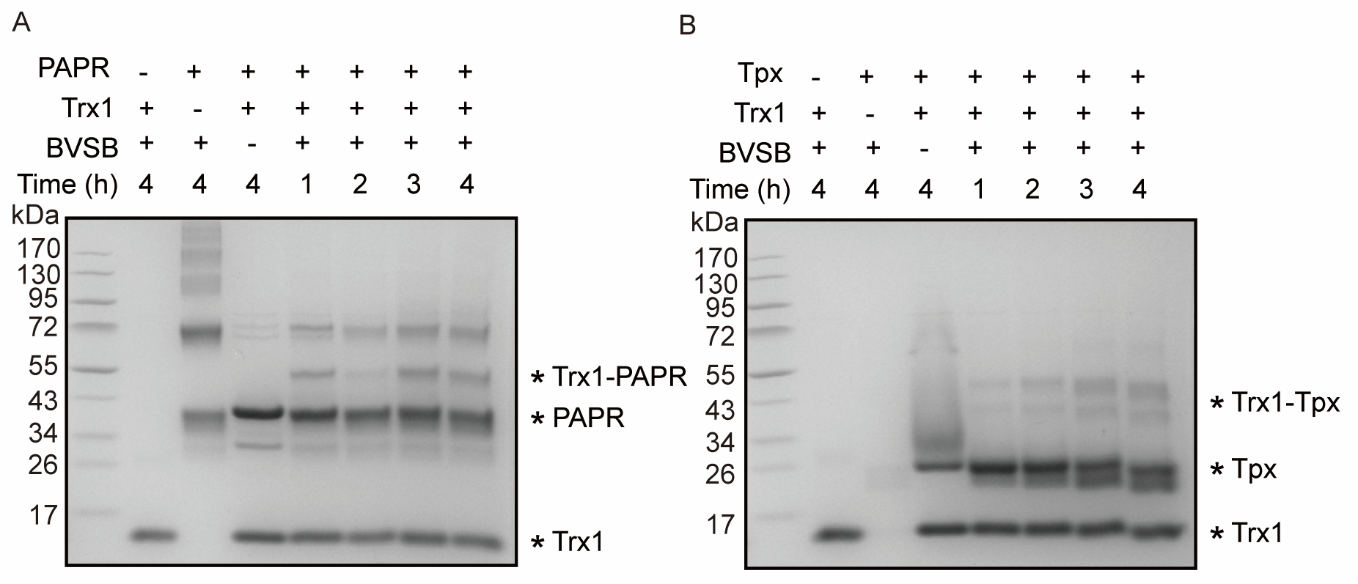


**Figure S8.** Optimization of BVSB cross-linking conditions. A) Trx1/PAPR complex was cross-linked with BVSB. B) Trx1/Tpx complex was cross-linked with BVSB.

**
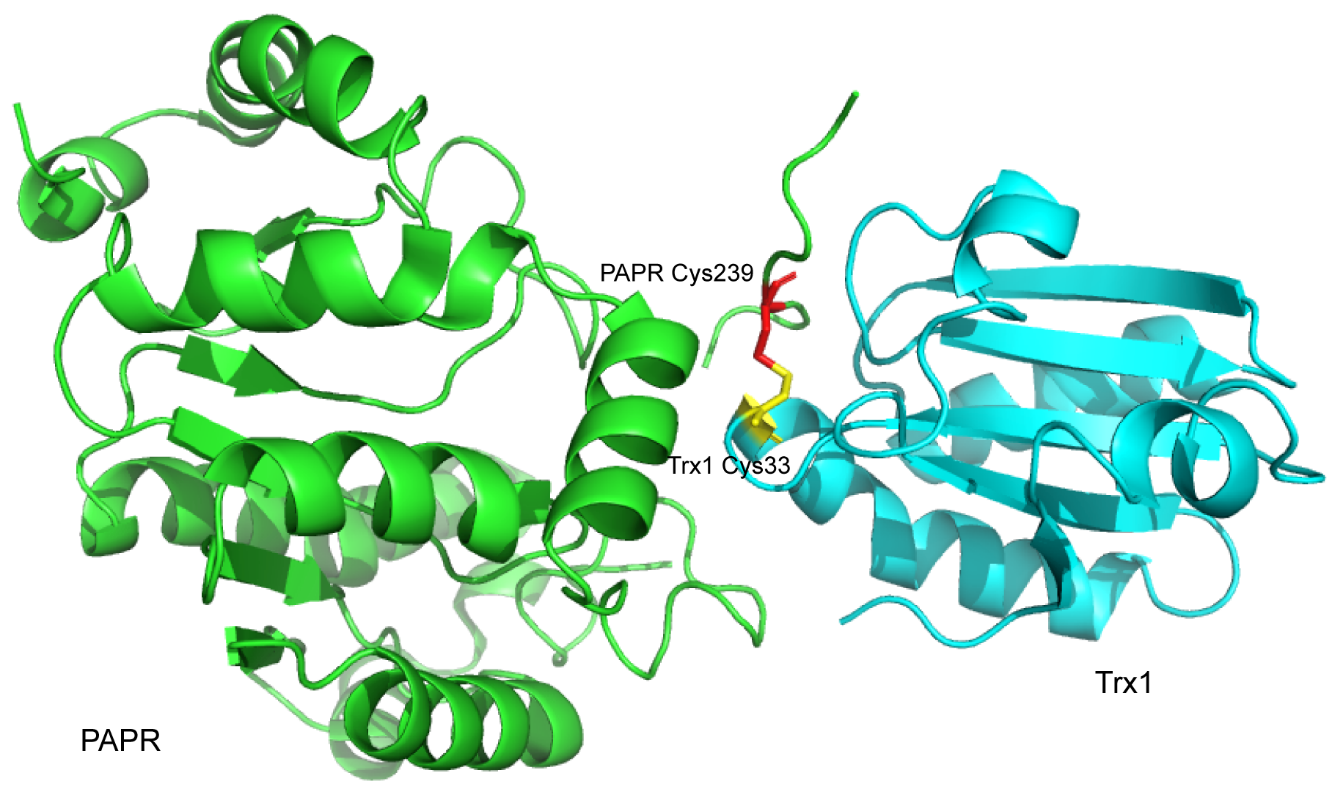
**

**Figure S9.** Trx1 Cys33 and PAPR Cys 239 in close position on protein structure (PDB ID 2O8V).

**
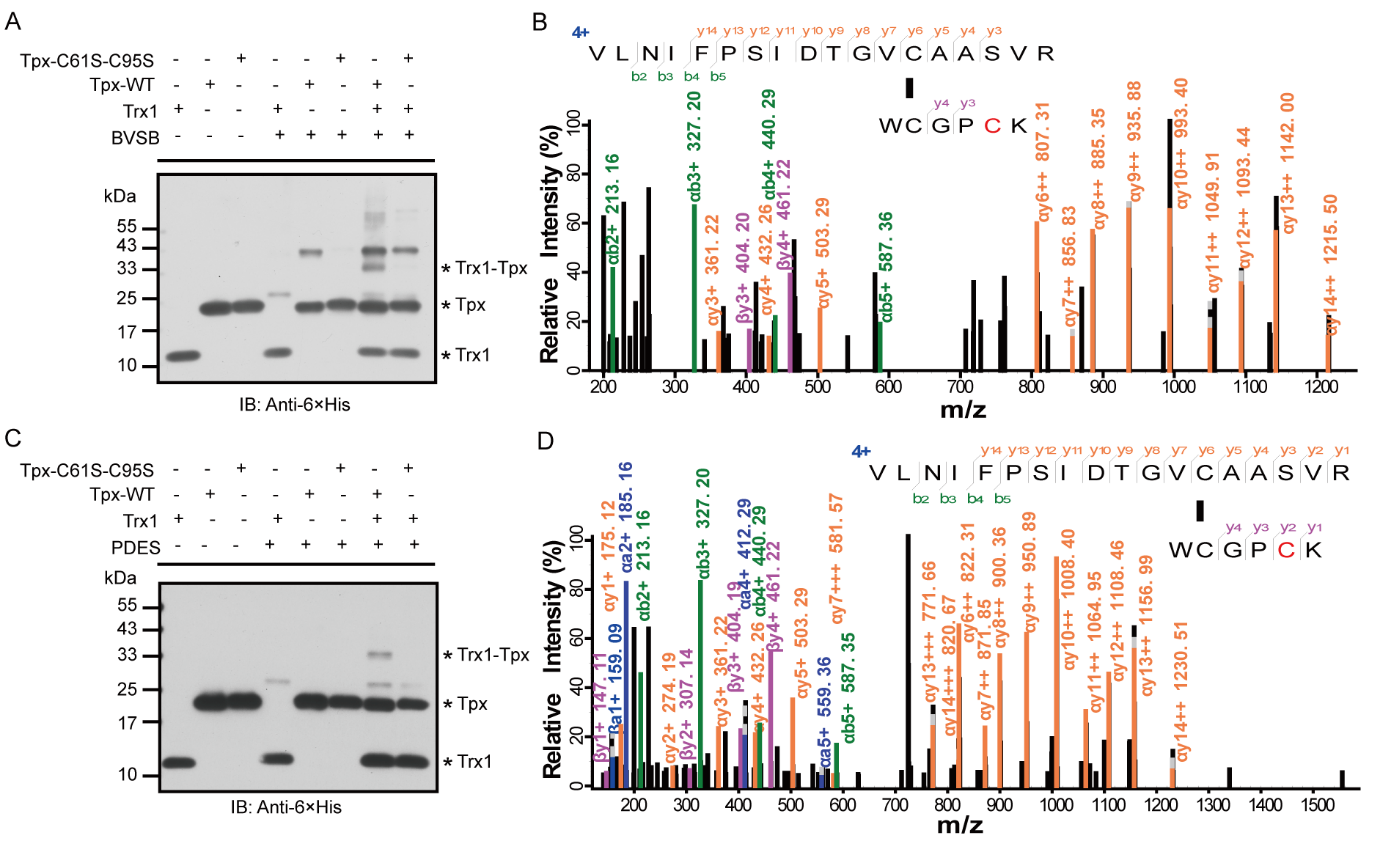
**

**Figure S10.** Cross-linking of Trx1 and Tpx with BVSB and PDES. A) Western blot analysis of *in vitro* BVSB cross-linking of Trx1 with Tpx and Tpx C61S/C95S mutant. B) Mass spectrum of BVSB cross-linking between Trx active site and Tpx active site. C) Western blot analysis of *in vitro* PDES cross-linking of Trx1 with Tpx and Tpx C61S/C95S mutant. D) Mass spectrum of PDES cross-linking between Trx active site and Tpx active site.

**
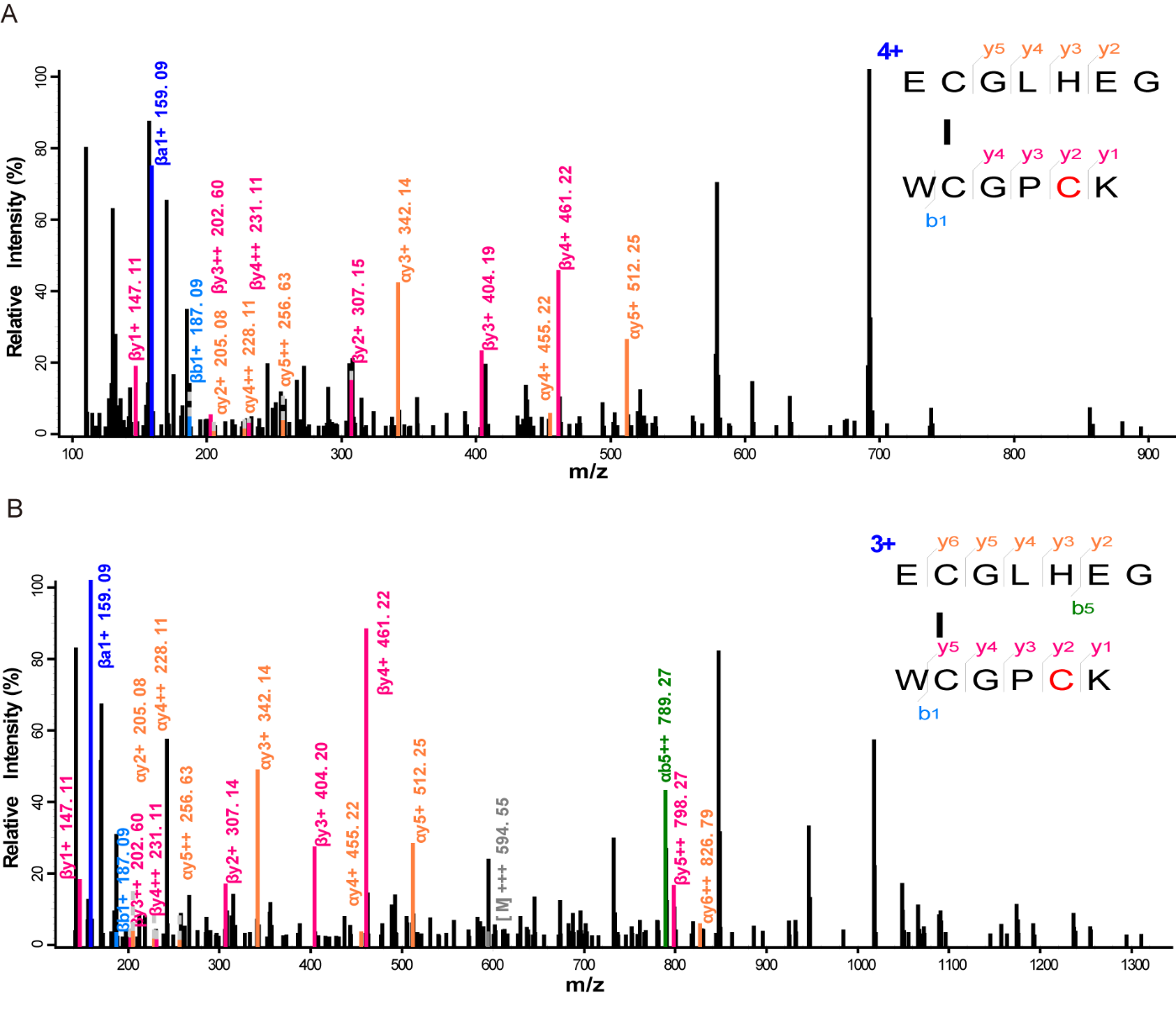
**

**Figure 11.** Cross-linking of Trx1/PAPR in live cells. A) Mass spectrum of BVSB live cells cross-linking between Trx active site and PAPR active site. B) Mass spectrum of PDES live cells cross-linking between Trx active site and PAPR active site.

**
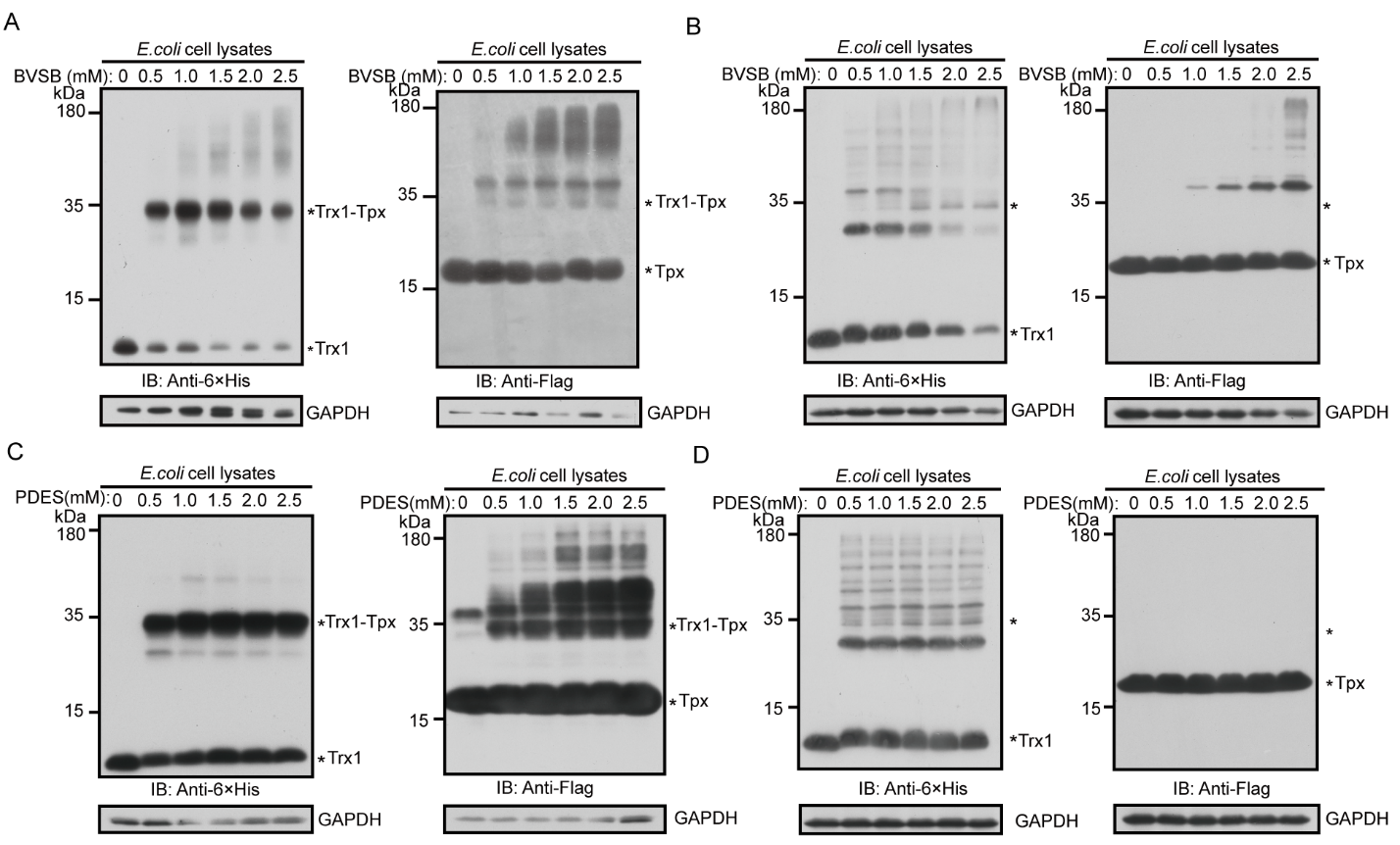
Figure S12.** Cross-linking of Trx1 and Tpx in live cells. A) Western blot of cell lysate of cells co-expressing Trx1 and Tpx, showing the *in vivo* cross-linking of Trx1 and Tpx by BVSB. B) Western blot of cell lysate of cells co-expressing Trx1 and TpxC61S/C95S mutant, cross-linking band of Trx1/Tpx heterodimer decreased. C) Western blot of cell lysate of cells co-expressing Trx1 and Tpx, showing the *in vivo* cross-linking of Trx1 and Tpx by PDES. D) Western blot of cell lysate of cells co-expressing Trx1 and and TpxC61S/C95S mutant, cross-linking band of Trx1/Tpx heterodimer decreased.

**
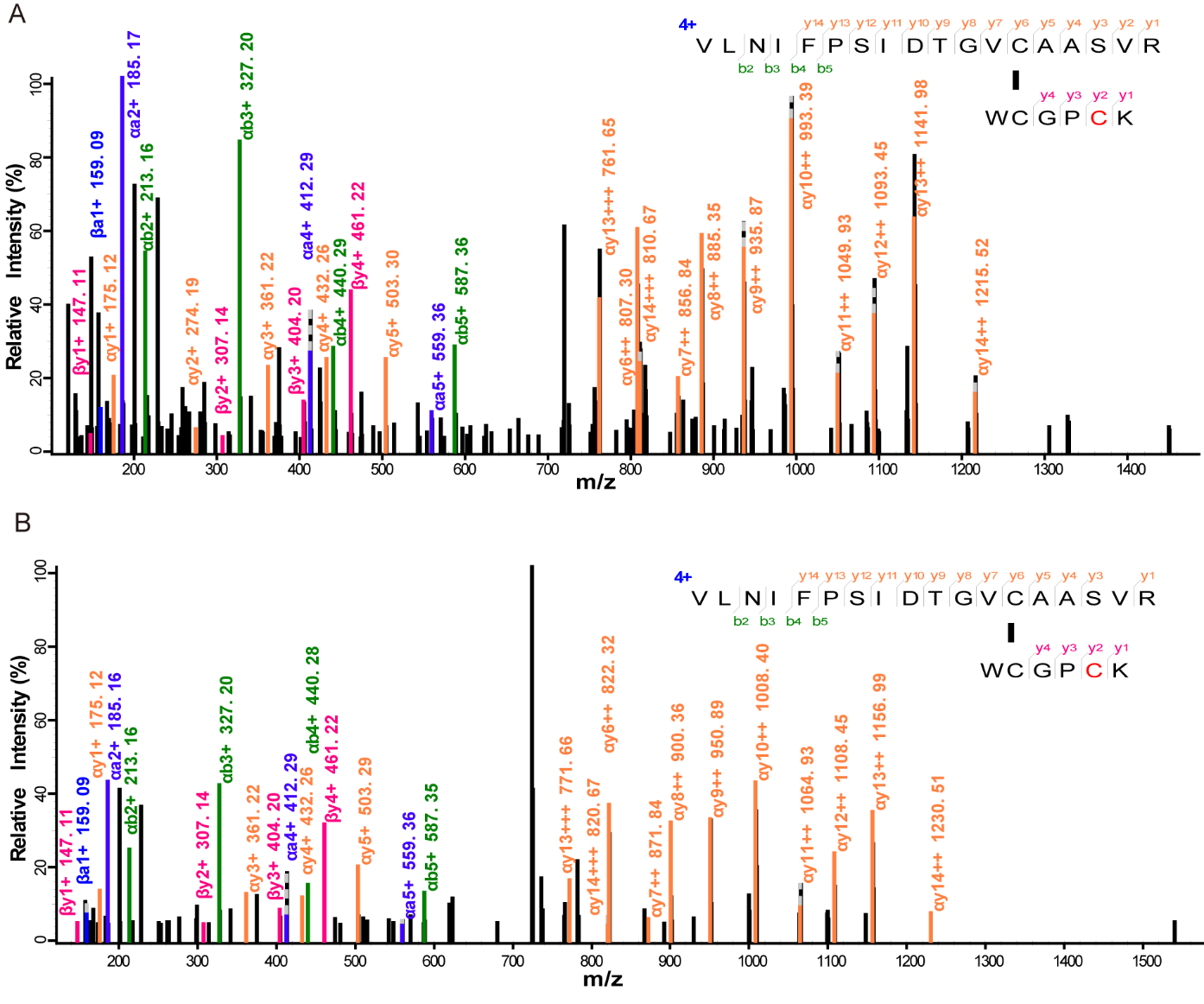
Figure S13.** Cross-linking of Trx1/Tpx in live cells. A) Mass spectrum of BVSB live cells cross-linking between Trx active site and Tpx active site. B) Mass spectrum of PDES live cells crosslin-king between Trx1 active site and Tpx active site.


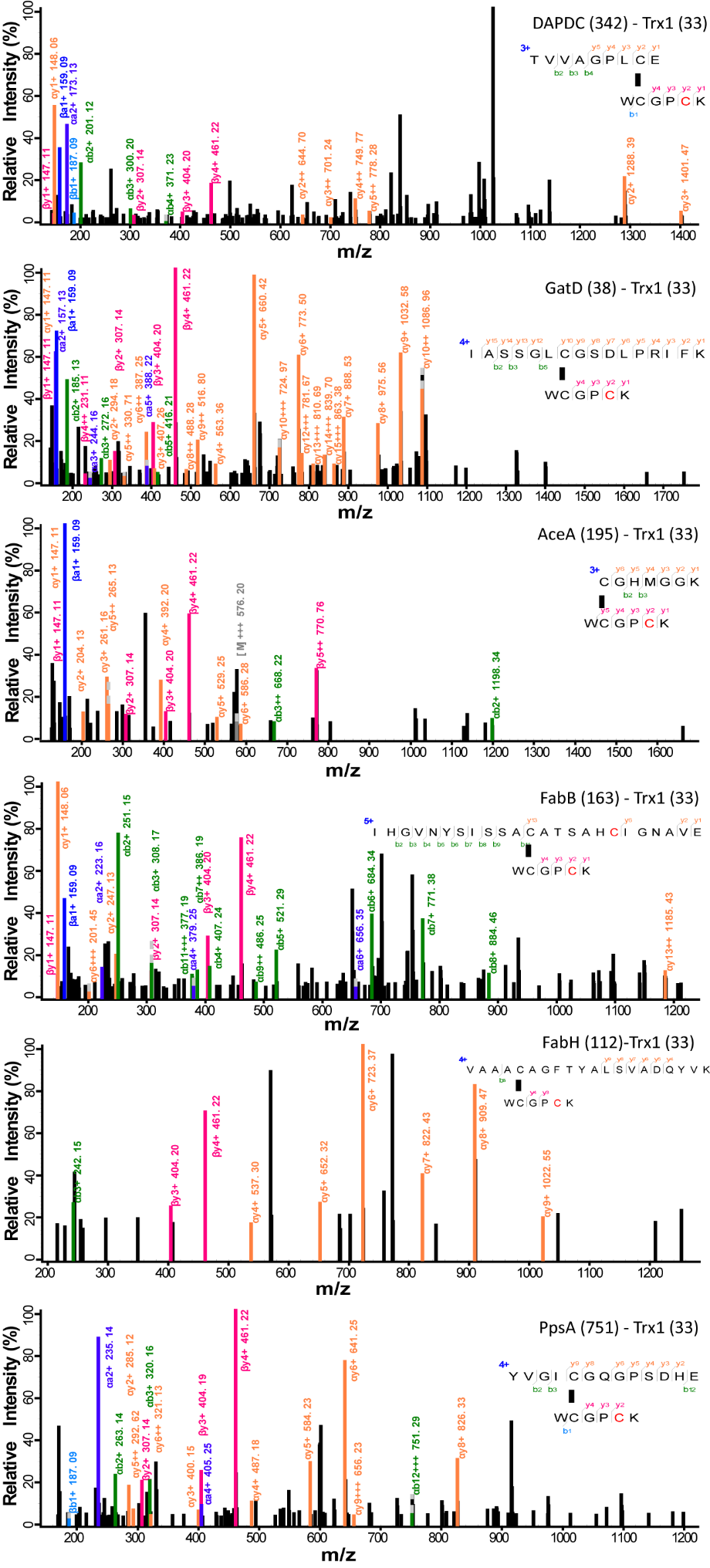


**Figure S14.** Mass spectra of Trx1 cross-linked with active sites of DAPDC, GatD, AceA, FabB, FabH, PpsA.


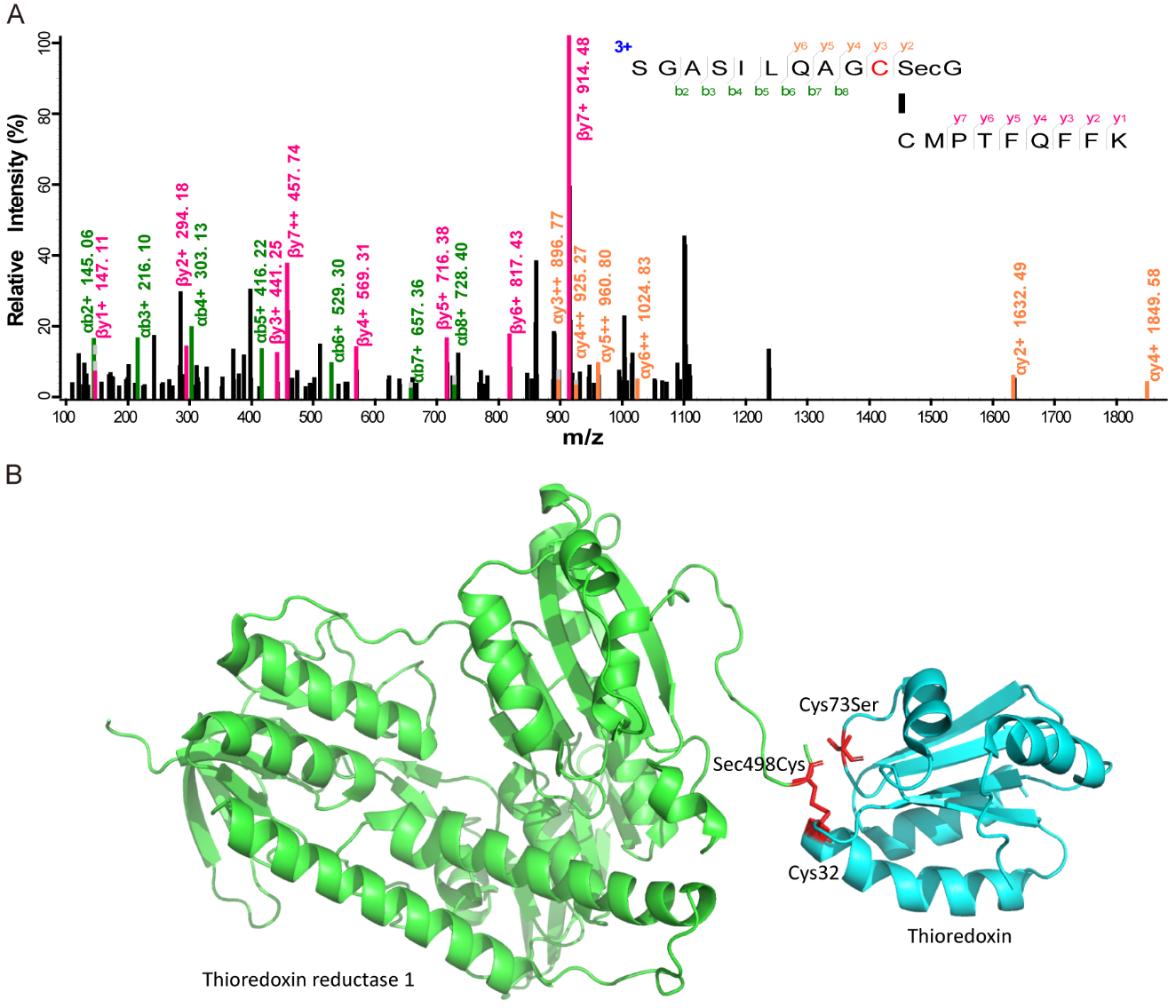


**Figure S15.** BVSB cross-links TXNRD1 (Sec648) with Trx1 (Cys73). A) Mass spectrum of TXNRD1 (Sec648) - Trx1 (Cys73) cross-linking. B) Trx1 (Cys73) is close to TXNRD1 (Sec648) on protein structure (PDB ID 3QFB).

**Figure S16**. Synthesis of SDBP.

**Figure S17**. ^1^H NMR spectrum of SDBP.

**Figure S18**. ^13^C NMR spectrum of SDBP.


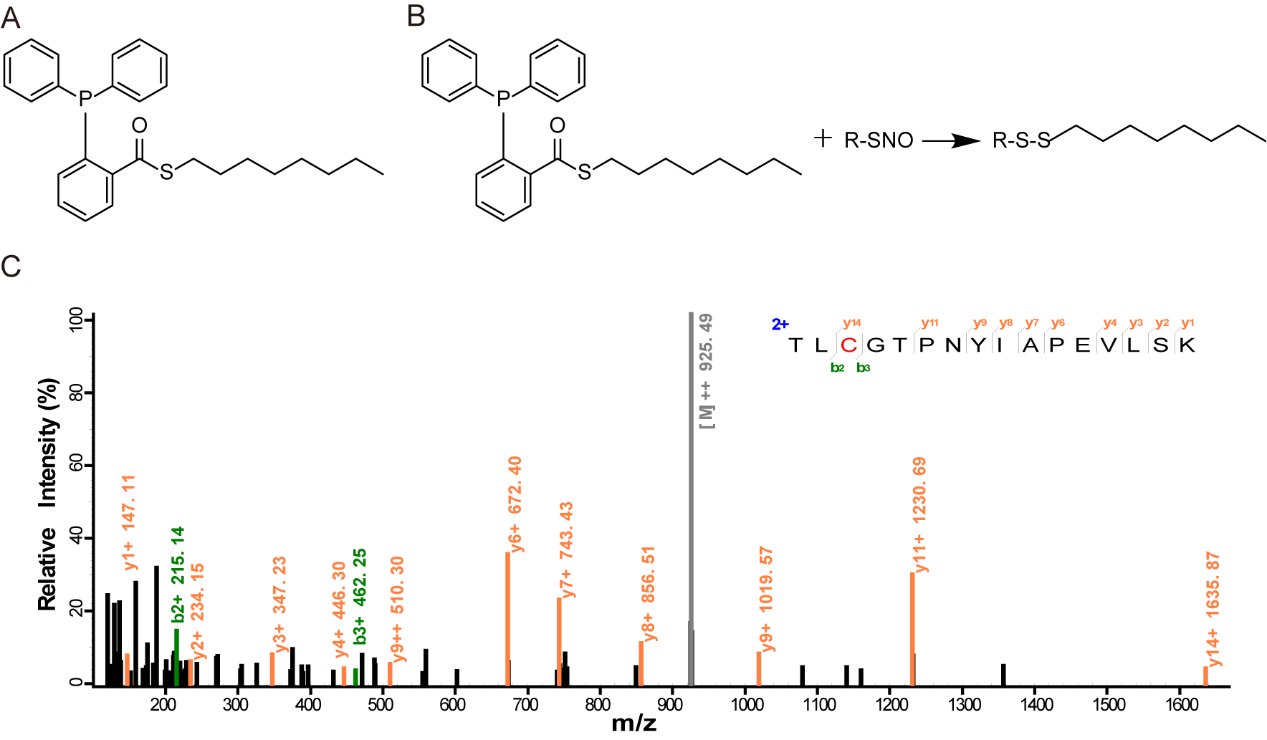


**Figure S19**. Validation of S-Nitrosylation site with phosphine compound. (A) Structure of SDBP. (B) Reaction of SDBP with S-Nitrosylation. (C) Spectrum of SDBP labelled peptide of PLK1

**2. Supplementary Notes**

Synthesis of cross-linkers of BVSB and PDES, and compound SDBP.

*General information of chemical synthesis*: All commercially available reagents and solvents (ACS grade) were purchased from commercial sources and used without further purification. Reactions were monitored by thin-layer chromatography (TLC) carried out on Merck silica gel 60 F-254 thin layer plates using UV light for visualization and an ethanolic solution of phosphomolybdic acid under heat or powdered iodine for developing. Flash column chromatography was generally performed on silica gel (200-300 mesh). The yields refer to chromatographically homogeneous materials. The ^1^H and ^13^C NMR spectra were recorded on a Bruker AV-500 or AV-400 spectrometer using CDCl_3_ or DMSO-*d_6_* as solvent. The chemical shifts (*δ*) are reported in *ppm* and coupling constants (*J*) in Hz. The following abbreviations were used to explain the multiplicities: s = singlet, d = doublet, t = triple. HRMS (High-resolution mass spectra) were conducted by a an Applied Biosystems Q-STAR Elite ESI-LC-MS/MS mass spectrometer under the condition of electrospray ionization (ESI).

*Chemical synthesis of BVSB:* BVSB can be synthesized by the synthetic route depicted in **Figure S1**. Briefly, tributyl(vinyl)tin (1395.2 mg, 4.4 mmol) was added slowly to a stirred solution of 1,3-benzenedisulfonyl chloride (550.2 mg, 2.0 mmol) in dry benzene (20 mL) at room temperature, then was allowed to heat at 60 ^o^C and stirred for 24 h. The reaction mixture was quenched by adding saturated aqueous NaHCO_3_ solution (10 mL) and extracted with ethyl acetate (100 mL). The combined organic extracts were washed with brine, dried over anhydrous Na_2_SO_4_, filtered, concentrated and purified by silica gel chromatography to give 320.3 mg (62% yield) BVSB as a white solid. ^1^H NMR (500 MHz, CDCl_3_) *δ* 8.39 (s, 1H), 8.15 (dd, *J* = 7.8, 1.4 Hz, 2H), 7.77 (t, *J* = 7.9 Hz, 1H), 6.67 (dd, *J* = 16.5, 9.6 Hz, 2H), 6.57 (d, *J* = 16.5 Hz, 2H), 6.17 (d, *J* = 9.6 Hz, 2H). ^13^C NMR (126 MHz, CDCl_3_) *δ* 141.81, 137.59, 132.79, 130.87, 130.06, 127.64. HRMS (ESI) *m*/*z*: [M + H]^+^ calcd for C_10_H_11_O_4_S_2_, 259.0093; found, 259.0067.

*Chemical synthesis of PDES:* PDES can be synthesized by the synthetic route depicted in **Figure S2**. Briefly, 2-chloroethanesulfonyl chloride (2037.5 mg, 12.5 mmol) and pyridine (1977.5 mg, 25.0 mmol) were added to a stirred solution of m-phenylenediamine (541 mg, 5.0 mmol) in CH_2_Cl_2_ (60 mL) at 0 ^o^C, then was allowed to warm to room temperature and stirred overnight. The reaction mixture was quenched by adding water and extracted with ethyl acetate (150 mL). The combined organic extracts were washed with brine, dried over anhydrous Na_2_SO_4_, filtered, concentrated and purified by silica gel chromatography to give 795.6 mg (55% yield) PDES as a white solid. ^1^H NMR (400 MHz, DMSO-*d_6_*) δ = 10.08 (s, 2H), 7.19 (t, *J*=8.1, 1H), 7.05 (s, 1H), 6.81 (dd, *J*=8.1, 1.8, 2H), 6.74 (dd, *J*=16.4, 9.9, 2H), 6.13 (d, *J*=16.4, 2H), 6.06 (d, *J*=9.9, 2H). ^13^C NMR (126 MHz, DMSO) δ = 138.58, 135.98, 129.73, 128.00, 114.46, 109.95. HRMS (ESI) *m*/*z*: [M + H]^+^ calcd for C_10_H_16_N_3_O_4_S_2_, 306.0577; found, 306.0573.

*Chemical synthesis of SDBP*: To a stirred solution of 2-(diphenylphosphaneyl)benzoic acid (505 mg, 1.65 mmol, 1.0 equiv) in dry DCM (6.0 mL) was added DCC (374 mg, 1.82 mmol, 1.1 equiv), DMAP (20 mg, 0.17 mmol, 0.1 equiv) and octane-1-thiol (266 mg, 1.82 mmol, 1.1 equiv) successively. The resulting mixture was stirred overnight at room temperature. The white solid formed in the reaction (1,3-dicyclohexylurea) was removed by filtration. The filtrate was concentrated under reduced pressure and purified by silica column chromatography (PE/EA) to give the desired product as thick yellow oil (540 mg, 75%). 1H NMR (500 MHz, CDCl3) δ 8.01 (dd, J = 7.3, 2.7 Hz, 1H), 7.40 (t, J = 7.2 Hz, 1H), 7.36 (t, J = 7.2 Hz, 1H), 7.23–7.34 (m, 10 H), 6.97 (dd, J = 7.2, 3.7 Hz, 1H), 2.96 (t, J = 7.4 Hz, 2H), 1.51 (m, 2H), 1.22–1.38 (m, 10 H), 0.88 (t, J = 6.9 Hz, 3H). 13C NMR (500 MHz, CDCl3) δ 192.8, 142.0, 141.8, 137.9, 137.8, 137.7, 137.6, 134.5, 134.0, 133.7, 131.6, 128.9, 128.8, 128.5, 128.4, 128.3, 31.8, 29.7, 29.2, 29.1, 28.9, 22.6, 14.1. HRMS (ESI) m/z: [M + H]+ calcd for C27H32OPS, 435.1906; Found: 435.1909.
